# Physiological and multiomics analyses on photosynthesis and carbon utilization reveal biochemical carbon-concentrating mechanisms in *Hygrophila difformis* under submergence

**DOI:** 10.1093/hr/uhag230

**Published:** 2026-06-15

**Authors:** Abeer Kazmi, Gaojie Li, Jingjing Yang, Xuyao Zhao, Md Fakhrul Islam, Xiaozhe Li, Dwi Fajar Sidhiq, Seisuke Kimura, Keiko U Torii, Hongwei Hou

**Affiliations:** 1State Key Laboratory of Breeding Biotechnology and Sustainable Aquaculture, Institute of Hydrobiology, Chinese Academy of Sciences, Wuhan 430072, Hubei, China; University of Chinese Academy of Sciences, Beijing 100049, China; 1State Key Laboratory of Breeding Biotechnology and Sustainable Aquaculture, Institute of Hydrobiology, Chinese Academy of Sciences, Wuhan 430072, Hubei, China; University of Chinese Academy of Sciences, Beijing 100049, China; 1State Key Laboratory of Breeding Biotechnology and Sustainable Aquaculture, Institute of Hydrobiology, Chinese Academy of Sciences, Wuhan 430072, Hubei, China; University of Chinese Academy of Sciences, Beijing 100049, China; 1State Key Laboratory of Breeding Biotechnology and Sustainable Aquaculture, Institute of Hydrobiology, Chinese Academy of Sciences, Wuhan 430072, Hubei, China; University of Chinese Academy of Sciences, Beijing 100049, China; 1State Key Laboratory of Breeding Biotechnology and Sustainable Aquaculture, Institute of Hydrobiology, Chinese Academy of Sciences, Wuhan 430072, Hubei, China; University of Chinese Academy of Sciences, Beijing 100049, China; 1State Key Laboratory of Breeding Biotechnology and Sustainable Aquaculture, Institute of Hydrobiology, Chinese Academy of Sciences, Wuhan 430072, Hubei, China; University of Chinese Academy of Sciences, Beijing 100049, China; Department of Industrial Life Sciences, Faculty of Life Sciences, Kyoto Sangyo University, Kyoto, Japan; Department of Industrial Life Sciences, Faculty of Life Sciences, Kyoto Sangyo University, Kyoto, Japan; Center for Plant Sciences, Kyoto Sangyo University, Kyoto, Japan; Institute of Transformative Bio-Molecules (WPI-ITbM), Nagoya University, Nagoya, Aichi, Japan; Howard Hughes Medical Institute, The University of Texas at Austin, Austin, TX, USA; Department of Molecular Biosciences, The University of Texas at Austin, Austin, TX, USA; 1State Key Laboratory of Breeding Biotechnology and Sustainable Aquaculture, Institute of Hydrobiology, Chinese Academy of Sciences, Wuhan 430072, Hubei, China; University of Chinese Academy of Sciences, Beijing 100049, China; Institute of Emerging Agricultural Technology, Shenzhen University of Advanced Technology, Shenzhen 518055, China

## Abstract

*Hygrophila difformis* is an ornamental amphibious plant that develops distinct terrestrial (shallow-serrated) and submerged (deep-lobed) leaf forms in response to environmental conditions. Although previous studies have reported bicarbonate (HCO₃^−^) utilization and highlighted the involvement of carbonic anhydrase in inorganic carbon uptake, the photosynthetic strategy and carbon assimilation pathway operating under submergence remain unclear. Here, we applied an integrated framework combining physiological measurements, enzyme activity assays, transcriptomic, proteomic, and metabolomic profiling, stable carbon isotope analysis, and ultrastructural observations to compare terrestrial and submerged leaves. Submerged leaves displayed a δ ^13^C value of −20.068‰, indicating a substantial shift toward C₄-like photosynthetic metabolism under aquatic conditions. Consistent with this signature, activities of key carboxylation and decarboxylation enzymes were significantly elevated, supporting a transition from C₃ photosynthesis toward an NAD-malic enzyme (NAD-ME)–type C₄ pathway. Multiomics analyses revealed extensive reprogramming of photosynthetic and starch metabolic networks in response to submergence, with strong upregulation of genes and metabolites associated with the NAD-ME–type C₄ cycle, including cytosolic aspartate aminotransferase and alanine aminotransferase, accompanied by coordinated downregulation of Calvin cycle components. By contrast, terrestrial leaves retained high Rubisco activity and strong expression of Calvin cycle genes, consistent with classical C₃ photosynthesis. Reverse-transcription quantitative PCR analyses demonstrated marked induction of carbonic anhydrase genes under submerged conditions, with HdαCA9 showing a 194-fold increase in transcript abundance, highlighting its central role in underwater carbon utilization. Transmission electron microscopy further revealed dimorphic chloroplasts within submerged mesophyll cells. Collectively, results demonstrate plasticity in *H. difformis* and provide insights into C₄-like carbon assimilation strategies in amphibious plants.

## Graphical Abstract

**Graphical Abstract:**
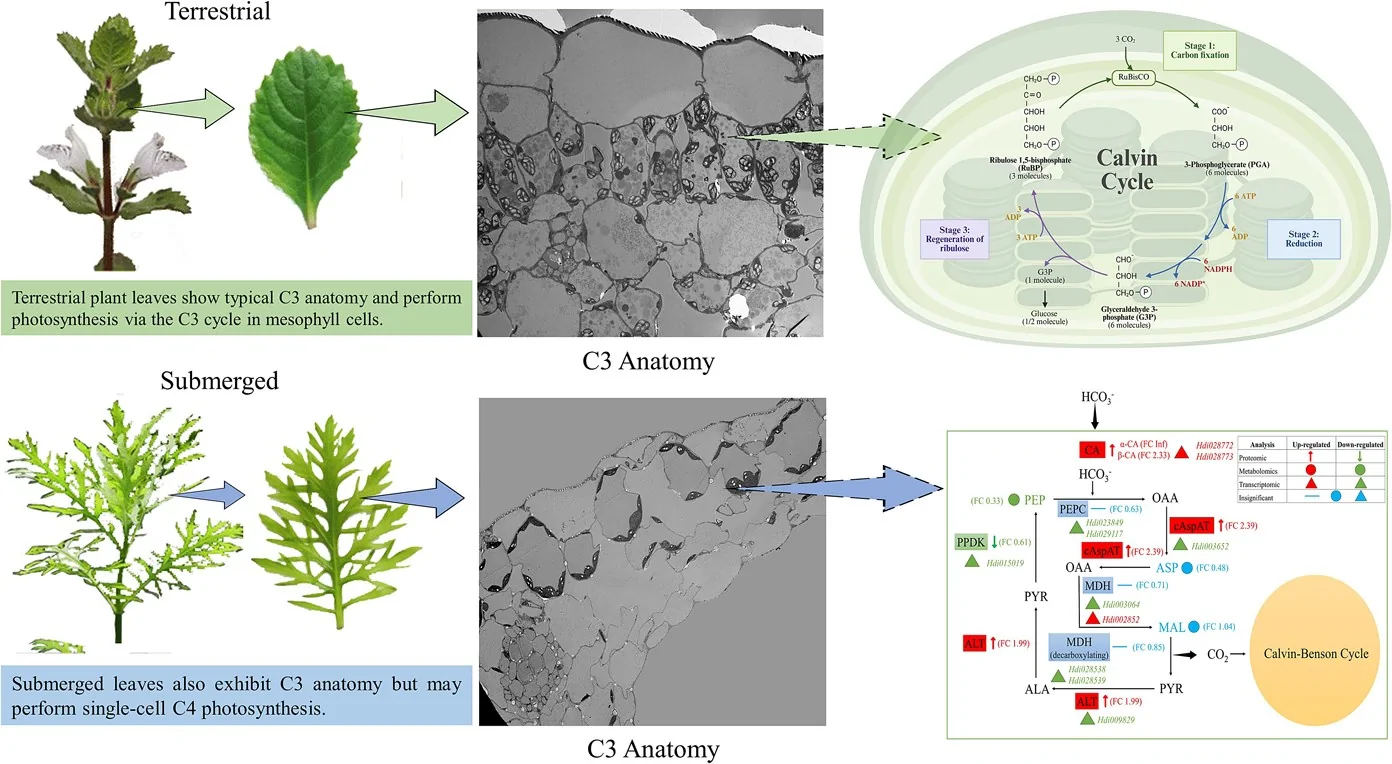

## Introduction

Submerged aquatic plants are essential components of freshwater ecosystems, offering critical ecological services such as oxygenation, nutrient cycling, and habitat provisioning [1]. However, their photosynthesis is often inhibited by the lack of CO_2_ due to high transport resistance, higher water pH, and the presence of an external boundary layer [2]. In most freshwater environments, bicarbonate (HCO₃^−^), rather than CO₂, dominates the pool of dissolved inorganic carbon (DIC) [3]. Consequently, many submerged macrophytes have evolved CO₂-concentrating mechanisms (CCMs) to increase the intracellular CO₂ concentration at the active site of ribulose-1,5-bisphosphate carboxylase/oxygenase (Rubisco) [4, 5]. These adaptations allow plants to elevate CO₂ concentrations at the active site of Rubisco, thereby minimizing photorespiration. Two principal HCO₃^−^ utilization strategies are commonly observed: one involves extracellular carbonic anhydrase (CAext) converting HCO₃^−^ into CO₂, which then diffuses into cells; the other entails direct HCO₃^−^ uptake via anion exchangers (AEs) in the plasma membrane [6, 7]. By facilitating the dehydration of HCO₃^−^ to CO₂ at the cell surface or within tissues, CA enhances the local availability of CO₂ for fixation by Rubisco, thereby supporting underwater photosynthesis and CO₂-concentrating mechanisms in submerged plants [8].

In addition to these biochemical mechanisms, some aquatic plants utilize specialized photosynthetic pathways such as C_4_ and crassulacean acid metabolism (CAM) to concentrate CO₂. Both pathways rely on phosphoenolpyruvate carboxylase (PEPC) for the initial carboxylation of HCO₃^−^, forming a four-carbon acid that is later decarboxylated to release CO₂ near Rubisco. While CAM separates carbon fixation temporally, C₄ metabolism separates it spatially and is typically associated with Kranz anatomy in terrestrial plants [9, 10]. Interestingly, aquatic species such as *Hydrilla verticillata, Ottelia alismoides*, and *Egeria densa* have demonstrated C₄ photosynthesis without Kranz anatomy, suggesting the occurrence of single-cell C₄ metabolism [11–13]. CAM and C₄ adaptations are relatively rare in aquatic environments, with ~9% and ~4% of species exhibiting these traits, respectively [14–17]. Nevertheless, their presence underscores the evolutionary plasticity of aquatic plants in coping with carbon-limited habitats. For instance, *O. alismoides* exhibits constitutive C₄ photosynthesis in mature leaves, independent of ambient CO₂ levels [7, 11, 18], whereas *H. verticillate* and *E. densa* initiate C₄-like traits in response to CO₂ limitation [12, 13, 19–21].

*Hygrophila difformis* is a fast-growing ornamental plant, which exhibits environmentally induced leaf dimorphism, producing broad laminar leaves under terrestrial conditions and finely dissected leaves when submerged [22, 23]. This morphological plasticity supports gas exchange and survival across variable environments. Previous research on *H. difformis* has not only provided quantitative insights into its underwater photosynthetic carbon acquisition but also revealed significant gaps in understanding its biochemical carbon-concentrating mechanisms. Under submerged conditions, the leaves of *H. difformis* utilize HCO₃^−^ as a primary source of inorganic carbon. Inhibitor experiments conducted at a pH of 8.3 demonstrated that both the external CA inhibitor acetazolamide (AZ) and the anion exchanger inhibitor 4,4′-diisothiocyanatostilbene-2,2′-disulfonate (DIDS) significantly reduce underwater photosynthetic oxygen (O₂) evolution [3]. This finding indicates that both external CA and a DIDS-sensitive HCO₃^−^ uptake pathway play important roles in facilitating the use of bicarbonate [3]. Comparative transcriptome analysis identified 20 CA-related transcripts in *H. difformis*, suggesting compartmentalized roles in apoplastic HCO₃^−^ conversion and cytosolic CO₂ supply [3]. Recombinant *Hdα-CA1* and *Hdβ-CA1* proteins exhibited dominant CO₂ hydration activity, consistent with facilitating intracellular CO₂ for Rubisco fixation in the submerged state [3]. Biochemical and anatomical comparisons further show that submerged leaves have higher underwater photosynthesis rates under low dissolved inorganic carbon and greater CA activity than terrestrial leaves [24].

Collectively, these studies confirm that *H. difformis* uses a biophysical carbon-concentrating mechanism (CCM) involving external CAs and bicarbonate uptake pathways to facilitate underwater photosynthesis. However, the biochemical CCM, which would involve transitions in the photosynthetic cycle such as shifts from typical C₃ to C₄ or C₄-like pathways and the concerted activity of photosynthetic enzymes like PEP carboxylase, Rubisco, and decarboxylases, remains unidentified in *H. difformis*, and key enzymatic kinetics and regulatory dynamics of such potential biochemical adaptations under submerged conditions have yet to be elucidated.

According to a recent study, the freshwater macrophyte *O. alismoides* possesses three distinct CCMs, including C₄ metabolism of the NAD-malic enzyme (NAD-ME) subtype and HCO₃^−^ utilization during the day, together with CAM at night, all in the absence of Kranz anatomy [25]. Despite reports of HCO₃^−^ utilization and single-cell C₄ photosynthesis in submerged macrophytes such as *Ottelia* spp.*, H. verticillata*, and *E. densa* [11, 13, 19], the biochemical basis underlying the adoption of comparable CCMs in *H. difformis* remains largely unknown.

We hypothesize that submerged leaves of *H. difformis* adopt a functionally integrated C₄-like carbon assimilation strategy in the absence of classical Kranz anatomy, representing a distinct form of photosynthetic plasticity under aquatic conditions. To test this hypothesis, we aimed to: (i) evaluate carbon fixation pathways by integrating δ^13^C isotopic signatures with the activities of key photosynthetic and carbon-concentrating enzymes [Rubisco, PEPC, PPDK, NADP-ME, NAD-ME, and carbonic anhydrase (CA)]; (ii) resolve submergence-induced reorganization of photosynthetic metabolism through integrated proteomic, metabolomic, and transcriptomic analyses; and (iii) systematically characterize the CA gene family with respect to phylogenetic relationships, chromosomal distribution, *cis*-regulatory elements (CREs), and synteny to elucidate its potential regulatory and evolutionary roles in aquatic carbon acquisition. Together, these approaches aim to unravel the photosynthetic strategies employed by *H. difformis* under aquatic conditions, offering new insights into amphibious plant plasticity and informing future strategies for enhancing crop resilience to submerged and low-CO₂ environments.

## Results

### Anatomical, morphological, and physiological analysis of *H. difformis* in submerged conditions

A terrestrial leaf of *H. difformis* exhibits a broad leaf form and a definite serrated margin (Fig. 1A). A more detailed SEM image of the terrestrial leaf epidermis reveals the stomata, which are equally distributed and slightly recessed (Fig. 1B). A terrestrial leaf cross-section seen under a transmission electron microscope (TEM) reveals a well-developed palisade mesophyll with densely packed chloroplasts that are required for efficient photosynthesis. A thick cuticle and rigid cell walls reduce water loss, which is critical for terrestrial plants (Fig. 1C).

**Figure 1:**
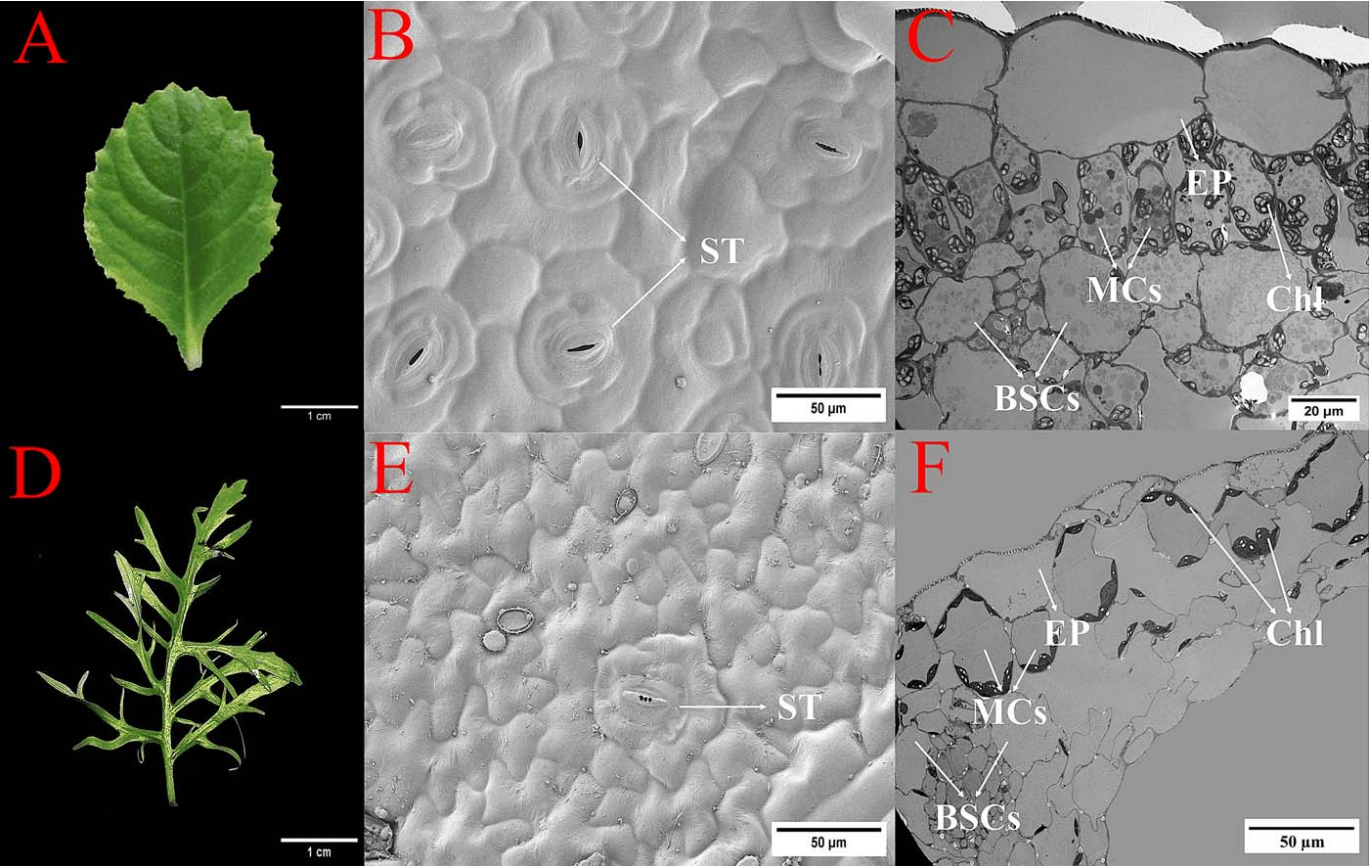
Morphological and anatomical features of the submerged and terrestrial leaf of *H. difformis.* (A) Morphology of terrestrial leaf, (B) Stomata of abaxial terrestrial leaf, (C) anatomy of terrestrial leaf using TEM, (D) Morphology of submerged leaf, (E) Stomata of abaxial submerged leaf, (F) anatomy of submerged leaf using TEM. ST, stomata; EP, epidermis; MCs, mesophyll cells; Chl, chloroplast; BSCs, bundle sheet cells.

In contrast, the general morphology of a submerged leaf is extensively dissected, with a thinner and more delicate structure than the terrestrial leaf (Fig. 1D). The SEM examination of the submerged leaf epidermis revealed fewer and less prominent stomata (Fig. 1E). The TEM analysis of the cross-section of the submerged leaf illustrates loosely packed mesophyll cells with substantial air spaces (Fig. 1F). The chloroplasts are more evenly distributed throughout the mesophyll.

We studied the morphological, physiological, and carbon isotope features of terrestrial and submerged leaves of *H. difformis* (Table 1). Submerged leaves of *H. difformis* tended to be slightly longer (4.02 ± 0.21 cm) and wider (3.17 ± 0.13 cm) than terrestrial leaves (3.51 ± 0.26 cm in length and 2.16 ± 0.16 cm in width); however, these differences were not statistically significant. Submerged leaves had a significantly higher dissection index (DI; 23.883 ± 0.827) than terrestrial leaves (4.942 ± 0.150). The greater DI in submerged leaves implies a more finely split structure, which improves their underwater photosynthesis and gas exchange efficiency. Submerged leaves had a higher total leaf area (11.67 ± 0.66 cm^2^) than terrestrial leaves (7.43 ± 1.16 cm^2^), allowing for better light acquisition and nutrient absorption. Terrestrial leaves had more thickness (0.316 ± 0.020 mm) than submerged leaves (0.126 ± 0.011 mm), resulting in less water loss and better mechanical stability against environmental stress. Terrestrial leaves had a greater leaf mass per area (LMA) (29.38 ± 1.996 g/m^2^) than submerged leaves (1.863 ± 0.901 g/m^2^).

**Table 1:** Morphological, physiological, and carbon isotope features of terrestrial and submerged leaves of *H. difformis*.

Type of data	Type of leaf		*P*-value
		Submerged	Terrestrial
Leaf length (cm)	4.02 ± 0.21	3.51 ± 0.26	0.057
Leaf width (cm)	3.17 ± 0.13^**^	2.16 ± 0.16	0.0011
Leaf area (cm^2^)	11.67 ± 0.66^**^	7.43 ± 1.16	0.0054
Dissection index (DI)	23.883 ± 0.827^***^	4.942 ± 0.150	< 0.0001
Leaf fresh weight (g)	0.025 ± 0.004^***^	0.103 ± 0.008	< 0.0001
Leaf dry weight (g)	0.0021 ± 0.0002^***^	0.0196 ± 0.00325	0.0005
Leaf thickness (mm)	0.126 ± 0.011^***^	0.316 ± 0.020	< 0.0001
Leaf mass per area (g/m^2^)	1.863 ± 0.901^***^	29.38 ± 1.996	< 0.0001
Chlorophyll-A content (mg/g)	0.2031 ± 0.0261^**^	0.3095 ± 0.0144	0.0038
Chlorophyll-B content (mg/g)	0.1269 ± 0.0192^***^	0.2979 ± 0.0149	0.0002
Total chlorophyll content (mg/g)	0.3301 ± 0.0321^***^	0.6075 ± 0.0272	0.0003
Carotenoid content (mg/g)	0.0617 ± 0.00285^**^	0.0845 ± 0.00438	0.0017
Hco_3_^−^ utilization ability (pH u/cm^2^)	0.1974 ± 0.0016^***^	0.0249 ± 0.0004	< 0.0001
Leaf δ13C (‰)	−20.068 ± 0.081^***^	−30.299 ± 0.1343	< 0.0001

The values presented are the mean ± standard error (*n* = 3). Asterisks indicate a significant difference (^*^ indicates P<0.05, ^**^ indicates P<0.01, and ^***^ indicates P<0.001).

Terrestrial leaves contain significantly more chlorophyll (A and B), and carotenoid content than submerged leaves (Table 1). Particularly, the concentration of chlorophyll-A in terrestrial leaves is 0.3095 ± 0.0144 mg/g, while chlorophyll-B is 0.2979 ±0.0149 mg/g. Submerged leaves exhibit 0.2031 ± 0.0261 mg/g of chlorophyll-A and 0.1269 ± 0.0192 mg/g of chlorophyll-B. Carotenoid content in terrestrial leaves is 0.0845 ± 0.00438 mg/g, whereas submerged leaves had 0.0617 ± 0.00285 mg/g. Furthermore, the total concentration of chlorophyll-A and chlorophyll-B in terrestrial leaves (0.6075 ± 0.0272 mg/g) is much higher than in submerged leaves (0.3301 ± 0.0321 mg/g).

In the current study, the isotopic composition of the leaves differs significantly between terrestrial and submerged leaves (Table 1). Terrestrial leaves possessed lower δ13C values (−30.299 ± 0.1343‰) than submerged leaves (−20.068 ± 0.081‰). Submerged leaves had higher δ13C values, suggesting a unique carbon consumption process tailored to submerged conditions with limited CO_2_ accessibility and slower diffusion.

### HCO_3_  ^−^ utilization ability of heterophyllous leaves of *H. difformis*

All pH drift assays were performed at 23°C under 60 μmol/m^2^/s white light flux. Under these conditions, submerged leaves exhibited a substantially higher HCO₃^−^ utilization capacity (0.19741 pH U/cm^2^) than terrestrial leaves (0.02499 pH U/cm^2^). This pronounced difference indicates that submerged leaves have a markedly enhanced capacity for bicarbonate-based carbon acquisition compared with terrestrial leaves when exposed to identical temperature and light conditions.

The comparison of photosynthetic activity of terrestrial and submerged leaves at different pH levels is shown in Fig. 2. Submerged leaves exhibit low activity at pH 6.3. In contrast, terrestrial plants exhibit vigorous photosynthesis, as demonstrated by the generation of bubbles, suggesting efficient CO_2_ use (Fig. 2A). Alternatively, at pH 8.3, terrestrial leaves show lower photosynthetic activity. In contrast, submerged leaves show increased activity, suggesting efficient bicarbonate (HCO_3_^−^) consumption. The data presented in Fig. 2B provide quantitative confirmation of these results. At pH 6.3, terrestrial leaves exhibit higher photosynthetic activity (~50 μmol O_2_ mg Chl^−1^ h^−1^), but submerged leaves perform much better at pH 8.3 (~65 μmol O_2_ mg Chl^−1^ h^−1^). This highlights the distinct pH optima for photosynthesis in terrestrial and submerged leaves, reflecting their respective environmental adaptations. Submerged leaves efficiently conduct photosynthesis at 8.3 pH because in alkaline conditions, bicarbonate becomes the major form of dissolved inorganic carbon (DIC).

**Figure 2:**
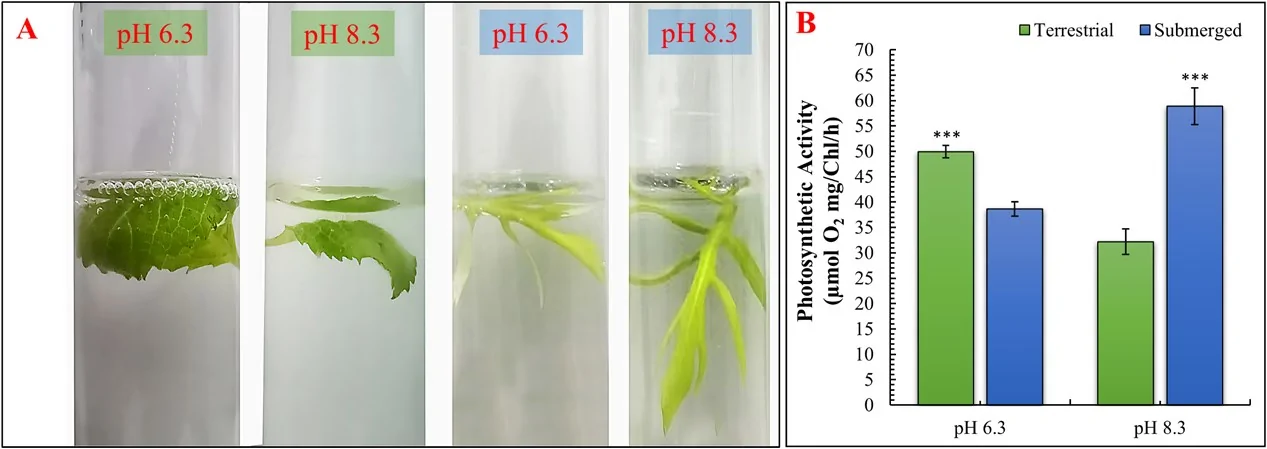
pH drift experiment to determine the HCO_3_^−^ utilizing ability. (A) Photosynthetic utilization of HCO₃^−^ in both terrestrial and submerged leaves of *H. difformis*. (B) The rate of underwater photosynthesis was assessed at pH 6.3 and pH 8.3. The medium comprised 10 mM NaHCO₃, 1.5 mM KCl, and 1.0 mM NaCl. Error bars represent ± SD (*n* = 3). Asterisks indicate a significant difference (^***^ indicates *P* < 0.001).

### Effects of inhibitors on photosynthetic activity and assessment of photosynthetic enzyme activity

Figure 3 depicts the impact of different inhibitors on photosynthetic activity in submerged leaves and compares the activity of photosynthetic enzymes in both submerged and terrestrial leaves of *H. difformis*. Different inhibitors were applied to submerged leaves to examine their effects on photosynthetic activity (Fig. 3A). The inhibitors included DIDS, an anion exchange protein inhibitor; Tris (hydroxymethyl) aminomethane (TRIS), an inhibitor of the bicarbonate/sodium symport; and acetazolamide (AZA) and ethoxyzolamide (EZ), which inhibit external and internal CA, respectively. In the absence of inhibitor treatment, the control group demonstrates the maximum photosynthetic activity, measuring around 60 μmol O₂ mg Chl^−1^ h^−1^. TRIS-treated leaves exhibit photosynthetic activity that is similar to the control group among the treated groups at around 55 μmol O₂ mg Chl^−1^ h^−1^, suggesting an insignificant impact on photosynthesis. AZA and DIDS treatments dramatically reduce photosynthetic activity to ~20 and 30 μmol O₂ mg Chl^−1^ h^−1^, respectively. EZ-treated leaves had photosynthetic activity of around 40 μmol O₂ mg Chl^−1^ h^−1^. These findings emphasize the importance of external CA and anion exchange in supporting photosynthesis in submerged leaves.

**Figure 3:**
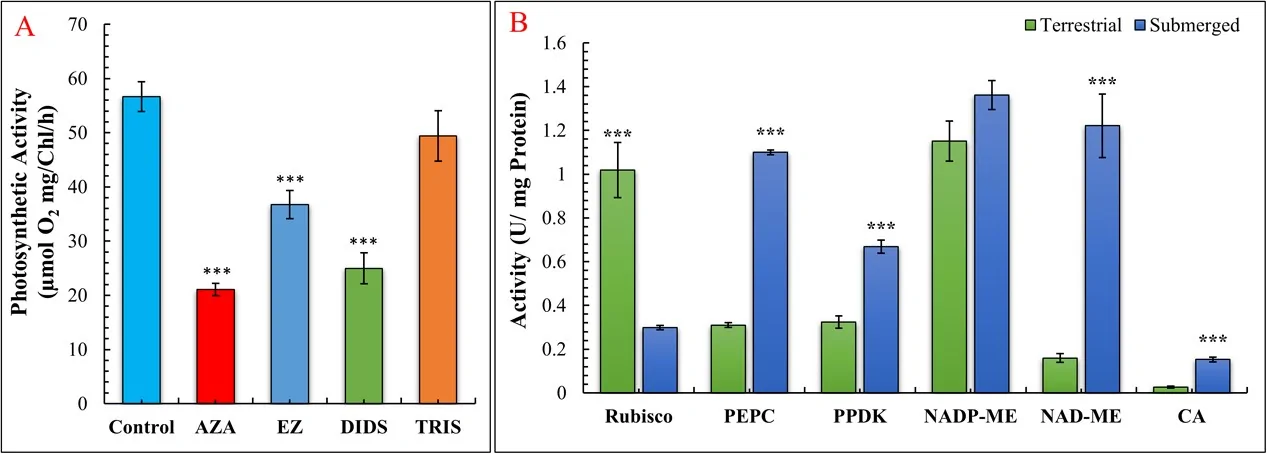
(A) Effect of different inhibitors on the photosynthetic activity of submerged leaves of *H. difformis*. Error bars represent ± SD (*n* = 3). Asterisks indicate a significant difference relative to control leaves (^***^ indicates *P* < 0.001). (B) Activity of photosynthetic enzymes (Rubisco, PEPC, and PPDK, NADP-ME, NAD-ME, and carbonic anhydrase) in the submerged and terrestrial leaves of *H. difformis*. Error bars represent ± SD (*n* = 3). Asterisks indicate a significant difference (^***^ indicates *P* < 0.001)

Figure 3B compares the activities of key enzymes involved in carbon fixation, decarboxylation, and inorganic carbon interconversion in terrestrial and submerged leaves of *H. difformis*. Distinct and coordinated variations in enzyme activities were observed between the two types of leaves, indicating significant metabolic reprogramming under submerged conditions.

Rubisco activity was significantly higher in terrestrial leaves (1.02 ± 0.13 U/mg protein) than in submerged leaves (0.30 ± 0.01 U/mg protein), reflecting a stronger reliance on direct CO₂ fixation via the Calvin–Benson cycle under aerial conditions. In contrast, submerged leaves exhibited markedly higher activities of phosphoenolpyruvate carboxylase (PEPC) (1.10 ± 0.01 U/mg protein) and pyruvate phosphate dikinase (PPDK) (0.67 ± 0.03 U/mg protein) compared with terrestrial leaves (PEPC: 0.31 ± 0.01 U/mg protein; PPDK: 0.32 ± 0.03 U/mg protein), indicating enhanced primary carboxylation of bicarbonate and regeneration of phosphoenolpyruvate in submerged tissues.

Activities of malic enzymes displayed contrasting patterns. NADP-malic enzyme (NADP-ME) activity showed no significant difference between terrestrial (1.15 ± 0.09 U/mg protein) and submerged leaves (1.36 ± 0.07 U/mg protein). In contrast, NAD-malic enzyme (NAD-ME) activity was dramatically elevated in submerged leaves (1.22 ± 0.15 U/mg protein) relative to terrestrial leaves (0.16 ± 0.02 U/mg protein). This pronounced induction of NAD-ME suggests that decarboxylation of C₄ acids in submerged leaves predominantly proceeds via an NAD-ME-dependent pathway.

CA activity was also significantly higher in submerged leaves (0.153 ± 0.010 U/mg protein) than in terrestrial leaves (0.027 ±0.005 U/mg protein), consistent with an enhanced capacity for rapid interconversion between CO₂ and HCO₃^−^ to support bicarbonate-based carbon acquisition under aquatic conditions.

### Multiomic analysis of submerged vs. terrestrial leaves of *H. difformis*

Multiomics analysis of *H. difformis* leaves indicates coordinated molecular adaptations to both submerged and terrestrial conditions. Proteomic results show 212 proteins upregulated and 329 downregulated in submerged leaves (SF 1A), with 42.7% localized in chloroplasts (SF 1B), indicating changes in photosynthesis and carbon assimilation. Heatmap analysis (SF 1C) reveals that upregulated proteins enhanced carbon-concentrating mechanisms, whereas downregulated ones are involved in stomatal regulation. The Kyoto Encyclopedia of Genes and Genomes (KEGG) classification and enrichment (SF 2A and B) show significant proteomic shifts in carbon metabolism, amino acid biosynthesis, oxidative phosphorylation, and stress-related pathways, with MAPK signaling downregulated.

Metabolomic analysis supports these findings, with 156 compounds upregulated and 381 downregulated in submerged leaves (SF 3A). Distinct group separation in Orthogonal Partial Least Squares Discriminant Analysis (OPLS-DA) (SF 3B) and differential clustering in the heatmap (SF 3C) indicate environment-driven metabolic reprogramming, particularly for flavonoids, terpenoids, and amino acids. KEGG pathway mapping (SF 4A) shows that metabolites are enriched in amino acid, carbohydrate, lipid, and secondary metabolic pathways. The differential abundance analysis (SF 4B) reveals significant variations in flavonoid biosynthesis, α-linolenic acid metabolism, photosynthesis, and hormone signaling.

In submerged leaves of *H. difformis*, the metabolite profile reflects a pronounced metabolic reprogramming associated with adaptation to low-CO₂, aquatic environments (Table S1). Lipid-related compounds such as LysoPC 22:6 and LysoPC 15:0 show pronounced accumulation, suggesting active membrane remodeling and lipid signaling under submerged conditions. Elevated levels of amino acid derivatives and small peptides (e.g. γ-Glu-Leu, Ile-Asn, Ile-Gly-Val, γ-Glu-Phe) indicate enhanced protein turnover and nitrogen recycling, supporting metabolic flexibility in low gas-exchange environments. Organic acids, including ethylmalonic acid, 2-methylsuccinic acid, and 4-hydroxy-2-oxopentanoate, reflect reprogramming of central carbon metabolism and energy balance. Additionally, increased alkaloids, phenolamines, and terpenoid derivatives point to activation of stress-response and defense pathways. Upregulation of nucleotide-associated metabolites further suggests elevated energy demand and nucleic acid metabolism. These metabolic changes highlight coordinated lipid restructuring, nitrogen reallocation, and stress adaptation mechanisms that facilitate submerged growth in *H. difformis*.

Transcriptomic profiling revealed broader regulatory alterations, with 3749 genes upregulated and 4032 downregulated under submerged conditions (SF 5A), along with noticeable group separation in PCA (SF 5B) and distinct expression clusters in the heatmap. According to the KEGG-based transcript annotation (SF 6A), the majority of genes are involved in metabolic (43.27%), environmental response (27.1%), and organismal system pathways (13.02%). The circular heatmap (SF 6B) further verifies condition-specific gene regulation, showing distinct activation and suppression patterns across pathways.

### Carbon concentrating mechanisms of submerged and terrestrial leaves

Figure 4 provides a proteomic analysis of the proposed C₄ photosynthetic pathway in *H. difformis* leaves, contrasting submerged and terrestrial growth conditions. The findings highlight the regulation of key enzymes in submerged leaves compared to terrestrial conditions, with fold changes (FCs) indicating upregulation, downregulation, or stability. Figure 4A illustrates a proposed model for bicarbonate (HCO₃^−^) transport into a plant cell and chloroplast. CO_2_ enters by the upregulated aquaporin PIP-type Ptom75 (FC = 1.89) and converts to HCO₃^−^ by β-CA (FC = 2.33). Extracellular HCO₃^−^ is proposed to be taken up by an unknown transporter, while α-CA (FC = ∞) may aid in conversion. The upregulation of these proteins highlights a possible HCO₃^−^ uptake mechanism and emphasizes the need to identify the unknown transporter involved.

**Figure 4:**
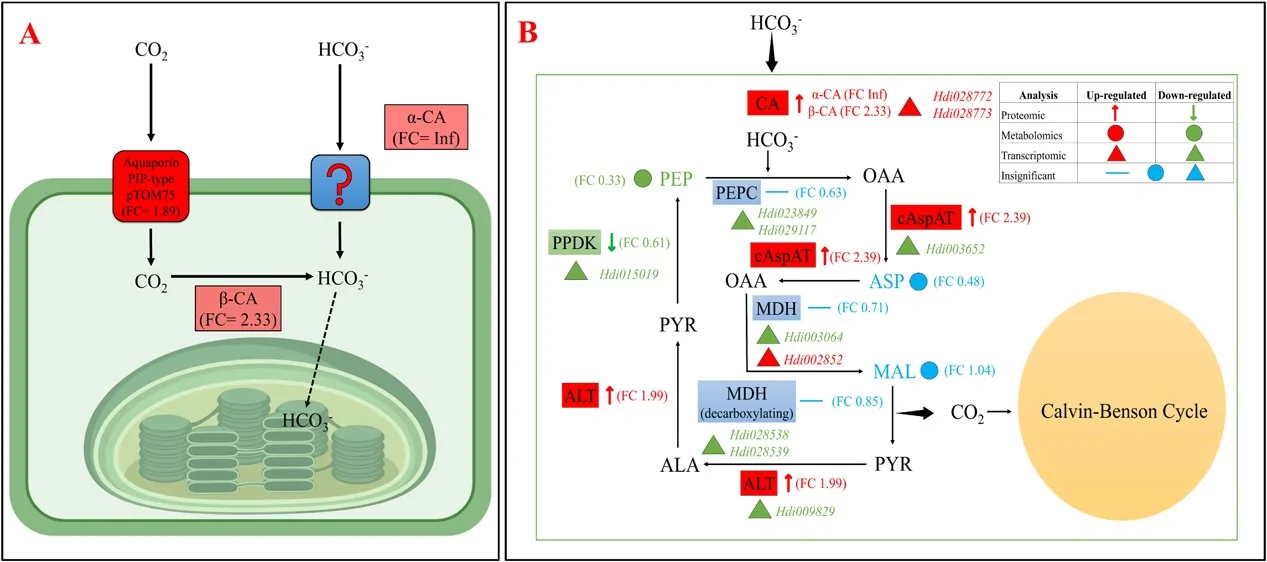
Modulation of the carbon fixation pathway in submerged *H. difformis*. (A) Hypothetical model of bicarbonate transport to the plant cell. (B) Proteomic data reveal increased activity of key enzymes, supporting a hypothesis that the shift toward C_3_–C₄ intermediate like photosynthesis plays an important role in carbon acquisition under submerged conditions. Fold changes (FCs) represent submerged vs. terrestrial conditions. FC inf = not detected in terrestrial leaves.

Figure 4B depicts the regulation of C₄-related carbon assimilation in submerged *H. difformis* based on integrated transcriptomic, proteomic, and metabolomic analyses. Consistent with the elevated CA activity observed in submerged leaves (Fig. 3B), both α-CA (FC = high, *Hdi028772*) and β-CA (FC = 2.33, *Hdi028773*) were strongly upregulated at the protein and transcript levels, indicating an enhanced capacity for rapid interconversion between HCO₃^−^ and CO₂. This molecular response corroborates the biochemical evidence for increased bicarbonate utilization under submerged conditions.

In contrast to the significantly higher PEPC and PPDK enzyme activities detected in submerged leaves (Fig. 3B), PEPC showed no significant change at the protein level and was downregulated at the transcript level (*Hdi023849, Hdi029117*) in the multiomics dataset, suggesting post-transcriptional regulation or enzyme activation rather than increased protein abundance. Similarly, PPDK was downregulated at the protein level (FC = 0.61; *Hdi015019*), accompanied by a marked reduction in phosphoenolpyruvate (PEP; FC = 0.33), indicating a constrained regeneration phase of the C₄ cycle despite elevated enzyme activity. This discrepancy highlights the flexible and condition-dependent regulation of C₄-related enzymes in submerged leaves. Cytosolic aspartate aminotransferase (cAspAT) was upregulated at the protein level (FC = 2.39), indicating increased transamination capacity, although its transcript (*Hdi003652*) was downregulated, which may explain the absence of a significant increase in aspartate content (FC = 0.48). Malate dehydrogenase (MDH) showed no significant change at the protein level (FC = 0.71), and its gene (*Hdi003064*) was downregulated, consistent with the relatively stable malate levels (FC = 1.04), despite upregulation of malate synthase (*Hdi002852*). Alanine aminotransferase (ALT) was significantly upregulated (FC = 1.99), suggesting enhanced carbon–nitrogen coupling in submerged leaves.

Multiomic analyses reveal a coordinated downregulation of the Calvin–Benson cycle in submerged leaves of *H. difformis* as compared to terrestrial (Fig. 5). Key enzymes such as ribulose-1,5-bisphosphate carboxylase/oxygenase large subunit (RbcL, FC = 0.37), phosphoribulokinase (PRK, FC = 0.60), ribose-5-phosphate isomerase (RpiA, FC = 0.58), and glyceraldehyde-3-phosphate dehydrogenase [GADH (NADP+), FC = 0.37] were significantly downregulated at the protein level, indicating reduced enzymatic capacity for C_3_ carbon fixation under submerged conditions. Transcriptomic results showed reduced expression of the genes encoding these enzymes, including *Hdi033233* (*RbcL*), and *Hdi019447*, *Hdi027779,* and *Hdi017829* (*PRK*), confirming transcriptional repression as a crucial regulatory mechanism. Metabolomic profiling confirms these findings, revealing reduced levels of essential Calvin cycle intermediates such as ribulose-5-phosphate (Ru5P), sedoheptulose-7-phosphate (S7P), and fructose-6-phosphate (F6P), which indicate diminished metabolic flux through the cycle during submersion. Interestingly, fructose-1,6-bisphosphatase (FBPase), encoded by *Hdi026316*, is upregulated at the protein level (FC =1.98), possibly as a compensatory mechanism to sustain carbon flux within the cycle. However, this upregulation is unlikely to fully counterbalance the substantial reductions in carbon fixation and RuBP regeneration. The pronounced downregulation of RbcL, GADH (NADP^+^), PRK, and RpiA collectively indicates that *H. difformis* significantly reduces its dependence on the C_3_ pathway under submerged conditions.

**Figure 5:**
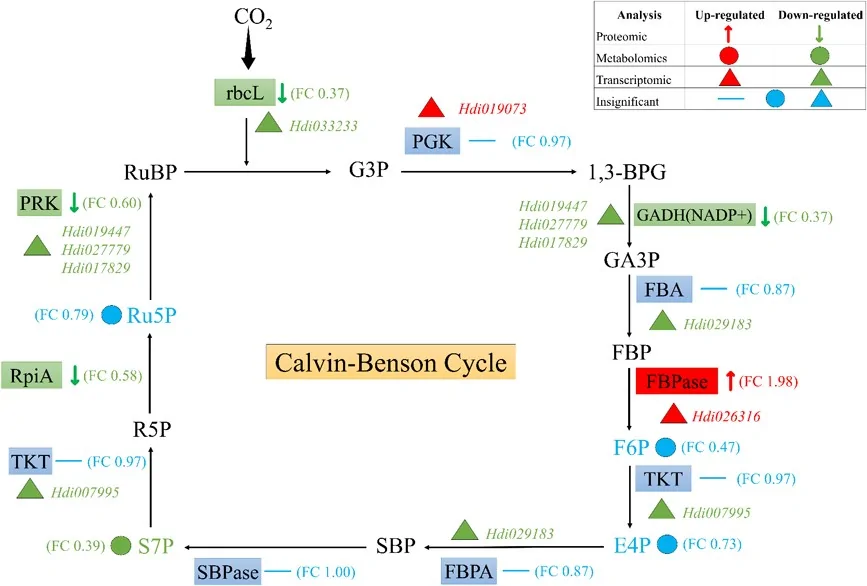
Regulation of the Calvin–Benson cycle in *H. difformis* leaves under submerged conditions compared to terrestrial conditions. Multiomic data reveal the downregulation of key enzymes [RbcL, PRK, RpiA, GADH (NADP+)], indicating a potential shift away from typical C_3_ carbon fixation during submersion. Fold changes (FCs) represent submerged vs. terrestrial conditions.

Taken together, the multiomics patterns in Fig. 4B support the enzyme activity data shown in Fig. 3B, indicating that submerged *H. difformis* operates a C₄-like carbon assimilation system characterized by enhanced bicarbonate conversion and decarboxylation capacity, while selectively downregulating energetically demanding regeneration steps. This flexible regulation, combined with the strong induction of NAD-ME activity at the biochemical level, is consistent with the operation of a NAD-ME-type C₄-like pathway optimized for carbon acquisition under submerged conditions.

### Starch metabolism and accumulation in the chloroplasts of submerged and terrestrial leaves of *H. difformis*

Supplementary Figure S7 comprehensively illustrates the alterations in starch metabolism in *H. difformis* under both terrestrial and submerged conditions, as observed through multiomic profiling and ultrastructural analysis. The pathway begins with glucose 1-phosphate, which is converted to ADP-glucose by ADP-glucose pyrophosphorylase (AGPase; *Hdi016164*) (SF 7A). Under submerged conditions, AGPase is significantly downregulated at the transcript level, while protein abundance remains insignificant. Downstream, granule-bound starch synthase (GBSS; *Hdi010080*) and debranching enzyme (DBE) were both significantly downregulated at the transcript and protein levels, indicating reduced starch biosynthesis. Starch branching enzyme (SBE; *Hdi001054*) and soluble starch synthase (SS; *Hdi027603*) also exhibited downregulation at the transcript level, although the protein level remained insignificant. In the starch degradation pathway, α-amylase (*Hdi031039, Hdi031060*) is significantly upregulated at the transcript level but downregulated at the protein level, indicating complex post-transcriptional regulation. Conversely, β-amylase (*Hdi029784*) is downregulated in transcriptomic results, while no significant change in protein abundance was observed.

The metabolomic analysis revealed a significant reduction in glucose levels, consistent with the overall inhibition of starch metabolism under submerged conditions.

The chloroplast of the terrestrial leaf of *H. difformis* is densely packed with large starch granules, indicating hyperaccumulation of starch (SF 7B). This observation aligns with the proteomic data, where key starch synthesis enzymes are either maintained or upregulated. Terrestrial leaves, being fully exposed to air and light, have better photosynthetic efficiency, which supports higher carbohydrate production and storage.

Supplementary Figure S7C shows a cell from a submerged leaf of *H. difformis* containing two distinct types of chloroplasts, without starch (SF 7D) and with starch (SF 7E). This heterogeneity suggests functional differentiation of chloroplasts under submerged conditions. The chloroplasts with starch may still retain photosynthetic activity or act as residual energy storage, while starch-lacking chloroplasts could be more specialized for other functions, such as nitrogen metabolism or adapting to low-light aquatic conditions.

### Genome-wide identification, characteristics, and expression patterns of the carbonic anhydrase family

To further understand how CAs support submerged photosynthesis, we identified CA genes in *H. difformis*. A BLASTp search on *Arabidopsis thaliana* CA sequences identified 21 putative CA genes, including 14 α-CA, 5 β-CA, and 2 γ-CA (Table S4). These isoforms differ in length (169–361 amino acids), molecular weight (18.56–41.03 kDa), and isoelectric point (pI 5.75–9.74). The majority have negative GRAVY scores, indicating their hydrophilic nature. Subcellular localization predictions suggest that these proteins are present in the cytoplasm, extracellular space, periplasm, and membranes. A phylogenetic study (SF 8) classified *HdCAs* as α, β, and γ classes. Each group contained CA isoforms from both *H. difformis* and *A. thaliana*, indicating a common evolutionary origin. Chromosomal mapping (Fig. 6A) identified five segmental duplications of *HdCA* genes. It suggests functional redundancy or divergence. A synteny analysis revealed gene-level conservation between *H. difformis* and other species. *Strobilanthes cusia* has the most *CA* gene pairs (24), followed by *A. paniculata* (21) and *A. thaliana* (16). Fewer pairings were found with monocots such as *Oryza sativa* (6), *Brachypodium distachyon* (4), and *Zea mays* (2). Stress and hormone-responsive motifs in CA gene promoters were identified using CRE analysis (Fig. 6B). Common elements were ABRE, TGACG motif, TCA element, MBS, and LTR. TCA and TGACG motifs were abundant in *HdαCA6,* indicating a potential function in stress response. The presence of ABRE in *HdβCA4* suggests abscisic acid regulation under drought or salinity. Reverse-transcription quantitative PCR (RT-qPCR) expression profiling (Fig. 7) revealed that six CA genes (*HdαCA3, HdαCA5, HdαCA6, HdβCA1, HdβCA4, HdβCA5*) were downregulated under submerged conditions. Three genes (*HdαCA9, HdαCA11, and HdβCA3*) were upregulated. *HdαCA9* exhibited a 194-fold increase, suggesting a crucial role in submergence adaptation.

**Figure 6:**
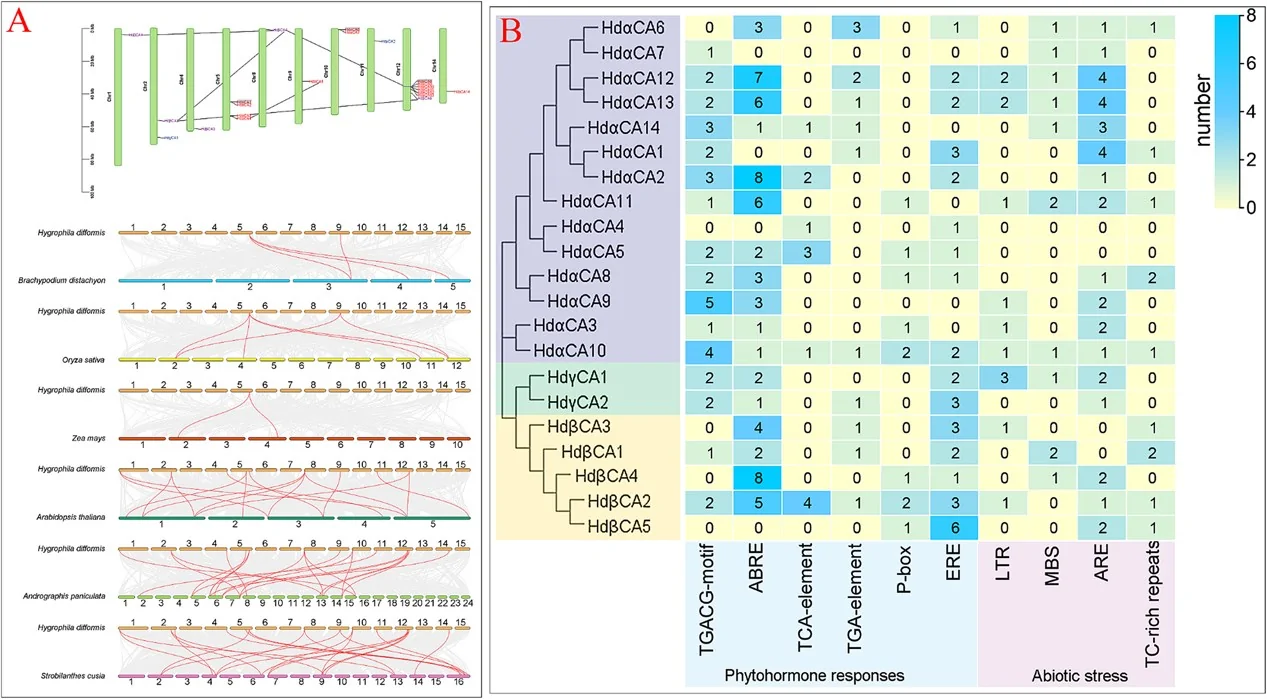
Characteristics and expression patterns of the carbonic anhydrase (CA) family. (A) Genomic distribution and synteny analysis of *carbonic anhydrase* (*CA*) genes in *H. difformis* across different chromosomes (top panel) and comparative synteny analysis between *H. difformis* and six other plant species (*B. distachyon*, *O. sativa*, *Z. mays*, *Arabidopsis thaliana*, *Andrographis paniculata*, and *S. cusia*) (bottom panels). The top panel displays the locations of *CA* genes on the chromosomes of *H. difformis*, with lines connecting segmental duplications. The bottom panels illustrate the conserved genomic regions between *H. difformis* and other species. (B) Heatmap analysis shows the distribution of *cis*-regulatory elements (CREs) in the *CA* gene promoter regions in *H. difformis*. The heatmap shows the presence of CREs associated with phytohormone responses and abiotic stress tolerance.

**Figure 7:**
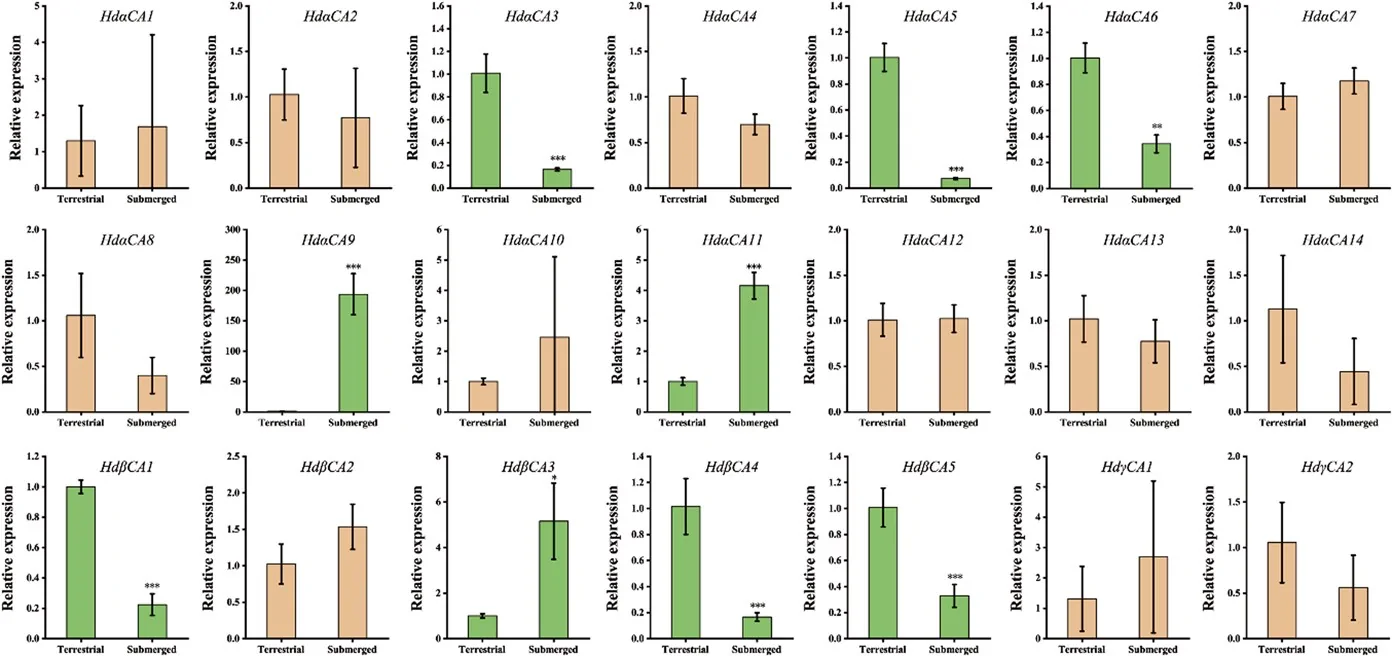
Relative expression of *CA* genes in *H. difformis*. Green bars indicate significant differences between terrestrial and submerged leaves. Orange bars indicate the nonsignificant difference between terrestrial and submerged leaves. Error bars represent ± SD (*n* = 3). Asterisks indicate a significant difference relative to terrestrial leaves (Student’s t-test: * indicates *P* < 0.05, ^**^ indicates *P* < 0.01, ^***^ indicates *P* < 0.001)

## Discussion

### C_3_ leaf structure is maintained across aquatic and terrestrial leaves

Seasonal fluctuations in water levels significantly alter environmental parameters such as light availability, dissolved oxygen, suspended particles, CO_2_, and nutrient levels [26]. These dynamic conditions, in turn, influence the growth patterns, biomass accumulation, and morphological adaptations of aquatic plants [27–29]. To cope with submerged environments, many aquatic plants exhibit specific morphological traits, such as thin leaves lacking stomata, a reduced cuticle, and narrow or dissected leaf shapes, adaptations that enhance gas exchange directly through the leaf surface [30–33].

*Hygrophila difformis* has a genome size of 871.92 Mb (Li *et al*., 2024). *Hygrophila difformis* has substantial variations between its aquatic and terrestrial leaves, making it an excellent model for studying heterophylly (Fig. 1).

Our morphological and anatomical findings are consistent with those of Horiguchi *et al*. [34] and Li *et al*. [31], who described that the submerged leaves of *H. difformis* are dissected and complicated, whereas the terrestrial leaves had a simpler structure. The submerged leaves are distinguished by their highly dissected morphology, limited cell layers with no obvious distinction between palisade and spongy tissues, profuse aerenchyma, and low stomatal density. In contrast, terrestrial leaves have weakly serrated borders, many cell layers with discrete palisade and spongy tissues, sparse aerenchyma, and a high stomatal density. These differences reflect the plant’s plasticity in adapting to varying environmental pressures.

The morphological divergence in *H. difformis* is driven by both environmental stimuli and endogenous phytohormonal regulation, underscoring the complexity of its adaptive strategy [31, 34]. Interestingly, comparative data from *H. polysperma*, a related species, showed that its submerged leaves are narrower and thinner but not dissected. Anatomical analysis indicated that chloroplasts were absent from the epidermal cells of both terrestrial and submerged leaves. While Kranz anatomy was present in the terrestrial leaves of *H. polysperma*, it was not present in the submerged leaves. Additionally, unlike certain C_4_ plants, the chloroplasts in the bundle sheath cells (BSCs) were not located close to the vascular bundles [35]. However, our results demonstrate that *H. difformis* leaves in both aquatic and terrestrial habitats have C_3_ anatomy, indicating significant variations in their physiological adaptations from this genus.

### Submerged and terrestrial leaves have different levels of photosynthetic pigments

The conversion of light into energy and the process of photosynthesis depend on photosynthetic pigments [36, 37]. Compared to previous studies that revealed higher chlorophyll b content in submerged leaves [7, 38, 39], our investigation found considerably higher concentrations of chlorophyll a, chlorophyll b, and total chlorophyll in terrestrial leaves (Table 1). This finding implies that in high-irradiance conditions, terrestrial leaves are more effective at absorbing light for photosynthesis.

Submerged leaves have lower photosynthetic pigment levels than terrestrial leaves due to various reasons [40]. The underwater environment has reduced light intensity and different light quality, owing to light dispersion and absorption by water and dissolved substances [41, 42]. Thus, submerged leaves have developed to optimize light absorption in these low-light environments. Chloroplasts are present in the top and lower epidermal cells of submerged leaves, which probably improves light absorption by increasing the surface area exposed to light [40, 42]. This adaptation is particularly beneficial in aquatic environments where low light intensity is common. Additionally, transcriptome research has shown that submerged leaves upregulate the expression of light-harvesting proteins, improving their ability to harvest light and adjust to the low-light conditions underwater [40]. This molecular adaptation enables submerged leaves to compensate for reduced pigment levels by improving the efficacy of their light-harvesting complexes.

In contrast, leaves on land have to balance the risk of photodamage against absorbing light as they are subjected to higher light intensities. This structural modification guards against oxidative stress and damage caused by excessive light, which might damage the photosynthetic apparatus. Furthermore, the content of carotenoids was nearly the same in both types of leaves, indicating a consistent protective function against photodamage across habitats. In addition to being accessory pigments in photosynthesis, carotenoids are essential for photoprotection because they absorb excess light energy as heat and scavenge reactive oxygen species, protecting the photosynthetic machinery from degradation [43].

### Physiological analysis revealed high efficiency of HCO_3_^−^ utilization in submerged leaves

A previous study has demonstrated that underwater photosynthesis in *H. difformis* relies on enhanced HCO₃^−^ utilization in submerged leaves at 25°C and 285 μmol m^−2^ s^−1^, supported by significant anatomical and biochemical acclimation [34]. Earlier studies demonstrated that submerged and submerged-terrestrial leaves exhibit higher underwater photosynthetic rates, elevated CA activity, and reduced cuticle and cell wall thickness, all of which facilitate inorganic carbon acquisition in an aquatic environment [3, 24, 34]. In contrast, terrestrially developed leaves were generally regarded as effectively CO₂-dependent and functionally incapable of bicarbonate use, as pH-drift assays typically detected no deviation from blank controls.

In this context, our pH-drift data add a new quantitative dimension to the understanding of inorganic carbon use in *H. difformis* by explicitly resolving area-normalized HCO₃^−^ utilization capacities for both leaf types under identical, low-irradiance conditions (23°C, 60 μmol m^−2^ s^−1^). We show that submerged leaves exhibit an ~8-fold higher bicarbonate utilization capacity than terrestrial leaves (0.19741 vs. 0.02499 pH U cm^−2^). Importantly, whereas previous studies treated terrestrial leaves as essentially nonusers of HCO₃^−^, our results reveal a small but measurable bicarbonate-driven pH change even in terrestrially formed leaves. This finding indicates that bicarbonate utilization in *H. difformis* is not a binary trait but instead represents a continuum, with a low basal capacity in terrestrial leaves that is strongly upregulated following submergence.

The analysis of photosynthetic activity at different pH levels reveals unique environmental adaptations in terrestrial and submerged leaves (Fig. 2). At pH 6.3, submerged leaves exhibit reduced photosynthetic activity. Meanwhile, oxygen bubble generation indicates effective CO_2_ use, demonstrating vigorous photosynthesis in terrestrial plants (Fig. 2A). In contrast, photosynthetic activity is reduced in terrestrial leaves and increased in submerged leaves at pH 8.3, indicating efficient consumption of bicarbonate (HCO_3_^−^). Our findings align with those of Horiguchi *et al*. [34], who reported that submerged leaves of *H. difformis* perform photosynthesis at a pH of 8.3 when grown at 25°C and exposed to a light intensity of 285 μmol m^−2^ s^−1^. In contrast, terrestrial leaves thrive at a pH of 6.3. Our results contradict the findings of Horiguchi *et al*. [35], who reported that submerged leaves of *H. polysperma* can perform photosynthesis at both pH 6.3 and 8.3.

The differences in pH optima for photosynthesis reflect unique environmental adaptations of the two leaf types. Terrestrial leaves can carry out photosynthesis more effectively because they are acclimated to conditions with high CO_2_ concentrations, usually at lower pH levels. The effective use of HCO_3_^−^ by submerged leaves at high pH emphasizes their adaptability to aquatic environments, where HCO_3_^−^ is converted to CO_2_ via CAs and DIDS-sensitive transporters, thereby maintaining photosynthetic activity.

Underwater photosynthesis mechanisms for HCO_3_^−^ utilization can be identified based on their sensitivity to different inhibitors. Previous studies employed four HCO_3_^−^ utilization inhibitors (AZA, TRIS, EZ, and DIDS) to distinguish these pathways in aquatic phototrophs [3, 7, 18, 34, 35, 44–48]. It was reported that the anion exchanger inhibitor DIDS inhibited high-DIC-affinity photosynthesis in the centric (*Chaetoceros muelleri*) and pennate diatoms (*Phaeodactylum tricornutum*). However, AZA (exogenous CA inhibitor) exhibited no such effect [48]. Furthermore, AZA reduced HCO_3_^−^ affinity in *Thalassiosira pseudonana* and *Cylindrotheca fusiformis*, though DIDS did not [48]. After being delivered into cells, HCO_3_^−^ is changed into CO_2_ by the DIDS-sensitive group. However, the DIDS-insensitive group promotes CO_2_ diffusion through external CA.

In the current investigation, submerged leaves of *H. difformis* demonstrated a higher affinity for HCO_3_^−^ than terrestrial leaves (Table 1). Furthermore, AZA (external CA inhibitor) and DIDS reduced the underwater net photosynthesis (Pn) of submerged leaves, although EZ and TRIS (an HCO_3_^−^/HC symporter inhibitor) did not (Fig. 2). Based on these data, *H. difformis* either uses an extracellular CA to convert HCO_3_^−^ to CO_2_ or uses a DIDS-sensitive transporter to transfer HCO_3_^−^ into its leaves. This HCO_3_^−^ utilization process is an efficient adaptation to submerged conditions. Our results contradict the findings of Horiguchi et al. [34], who concluded that EZ alone significantly reduced the photosynthetic activity of submerged *H. difformis* leaves, but AZA had no inhibitory impact. Although our experimental and growth conditions differed from those used by Horiguchi *et al*. [3], our results are highly consistent with their findings. Horiguchi *et al*. [3] demonstrated that both acetazolamide (AZA) and DIDS markedly inhibited the photosynthetic activity of submerged leaves of *H. difformis* when measurements were conducted at 25°C under a light intensity of 285 μmol m^−2^ s^−1^, with plants germinated under a 16 h dark/8 h light photoperiod. In contrast, our pH drift experiments were performed at a lower temperature (23°C) and substantially lower irradiance (60 μmol m^−2^ s^−1^). Despite these differences, the inhibitory effects observed in our study closely align with their conclusions, reinforcing the robustness of the underlying mechanism. Importantly, Horiguchi et al. [3] proposed that, under submerged conditions, photosynthesis in *H. difformis* is facilitated through the coordinated action of external CA and an as-yet-unidentified HCO₃^−^ transporter, a mechanism that is strongly supported by our findings. In a variety of algae to higher plants, DIDS inhibits HCO_3_^−^ uptake and underwater photosynthesis [45, 49, 50]. Similarly, under submerged conditions, AZA or DIDS were reported to impede photosynthesis in *O. cordata* [18], *O. ovalifolia* [7], and *H. polysperma*. Huang *et al*. [49] reported the first direct HCO_3_^−^ uptake via a DIDS-sensitive SLC4 transporter in *O. alismoides*.

### Terrestrial leaves execute C_3_ photosynthesis, while submerged leaves shift toward the NAD-ME type of C₄ photosynthesis

Photosynthesis-related enzyme activities and carbon C13 isotope analysis were employed to get a better understanding of the photosynthetic pathways of *H. difformis* in both submerged and terrestrial conditions. The δ13C levels differed significantly between terrestrial and submerged leaves (Table 1). Terrestrial leaves exhibited lower δ13C values (−30.299 ± 0.1343‰). It is important to understand that atmospheric CO_2_’s δ13C is often maintained at −8‰. When atmospheric CO_2_ is the only carbon source used by plants for photosynthesis, the range of δ13C for C_3_ plants is −21‰ to −37.5‰, whereas the range for C_4_ plants is −10‰ to −17‰ [7, 51, 52]. Previously published data on 478 C_3_ species from 187 distinct areas in China show a range of δ13C from −33.50‰ to −22.00‰, with a mean of −27.10 ± 1.70‰ [53]. The findings provide credence to our findings, demonstrating that terrestrial *H. difformis* leaves execute C_3_ photosynthesis. Submerged leaves exhibited higher δ13C levels (−20.068 ± 0.081‰). This suggests a distinct carbon utilization method appropriate for submerged conditions with reduced CO_2_ accessibility and slower diffusion. However, based only on the δ13C result, it is not possible to deduce that submerged leaves possess a C_4_ pathway. There is no specified range of δ13C values for single-cell C_4_ photosynthesis. However, the δ13C value for single-cell C_4_ photosynthesis conducting Hydrilla leaves was recorded as −23.5‰ [54, 55].

Underwater photosynthesis in amphibious and aquatic macrophytes is increasingly recognized as being supported by diverse CCMs, many of which operate at the single-cell level and integrate biochemical C₄-like cycles, CAM, and HCO₃^−^ utilization in the absence of Kranz anatomy. Despite this growing recognition, quantitative comparisons of carboxylation and decarboxylation activities between heterophyllous leaf types remain limited. In *H. difformis*, earlier studies demonstrated that submerged leaves exhibit higher CA activity and greater underwater photosynthetic capacity than terrestrial leaves [3, 24, 34]. However, whether submergence is accompanied by enzyme-level reprogramming of a C₄-like cycle remained unresolved.

Our results provide clear evidence of such biochemical reorganization. Terrestrial leaves displayed higher Rubisco activity, consistent with predominant C₃-based CO₂ fixation in air, whereas submerged leaves showed pronounced upregulation of PEPC, PPDK, NAD-ME, and CA, together with reduced Rubisco activity. This coordinated enzyme profile indicates a shift toward a biochemical, NAD-ME–biased C₄-like module that is tightly coupled to HCO₃^−^ acquisition under submerged conditions. This pattern parallel to the pathway reported for submerged *Ottelia* species. In *O. alismoides*, high PEPC and PPDK activities, elevated NAD-ME activity, constitutive C₄-like metabolism, and efficient HCO₃^−^ use occur in the absence of Kranz anatomy [56]. Recent single-cell analyses further revealed that an NAD-ME–type C₄ pathway can be integrated within a single cell with daytime HCO₃^−^ utilization and nocturnal CAM through temporal regulation and isoform-specific deployment of key enzymes and transporters [25]. Our finding that submerged *H. difformis* leaves preferentially induce NAD-ME, while NADP-ME remains unchanged, complements these observations and suggests that NAD-ME–dominated decarboxylation represents a recurrent biochemical solution for single-cell, aquatic C₄-like CCMs. Notably, in *H. difformis*, this solution is expressed across contrasting heterophyllous leaf morphs rather than within a single leaf type.

In contrast, studies on *H. polysperma* indicate a divergent amphibious strategy: terrestrial leaves exhibit proto-Kranz-like C₄ features with elevated C₄-associated proteins, whereas submerged leaves downregulate C₄ components and rely more heavily on C₃ photosynthesis coupled with biophysical HCO₃^−^ use, as reflected by declining PEPC/Rubisco ratios under water [35]. Our findings, therefore, delineate an alternative amphibious trajectory in which submergence reinforces, rather than relaxes, biochemical CCM expression. By quantitatively positioning *H. difformis* along the emerging continuum of CCM strategies from traditional terrestrial C₄ lineages to single-cell NAD-ME C₄ combined with CAM systems in Ottelia, our data highlight that amphibious heterophylly can involve reconfiguration of C₄-related biochemistry, similar to subtypes. This process works alongside CA and transporter-mediated HCO₃^−^ uptake. As a result, our findings expand the known diversity and adaptability of aquatic CCM integration and emphasize submergence-driven biochemical specialization as a crucial aspect of photosynthetic adaptation in amphibious plants.

Freshwater monocots such as *H. verticillata*, *E. densa*, *S. subulata*, and *O. viscida* exhibit facultative single-cell C₄ photosynthesis [51, 54, 57–62]. Hydrilla thrives underwater, promoting C₄ photosynthesis under warm temperatures, low CO₂ levels, and intense light [21]. According to Bowes [54], this transformation is possible even in the absence of new leaf production. In *Hydrilla verticillata,* Rubisco and PEPC reside in the chloroplast and cytosol, respectively [21]. Despite early skepticism, the single-cell C₄ system in *H. verticillata* currently has substantial supporting evidence. Similar to terrestrial C₄ species, Hydrilla exhibits enhanced expression and activity of C₄ enzymes during the activity of the C₄ cycle between the cytosol and chloroplast. Additionally, there is an increase in CA activity [54, 63, 64]. *Egeria densa* is also a facultative C₄ aquatic plant that converts to C₄ in high temperatures and certain light conditions [19, 51, 61, 65].

### Unveiling submerged survival: multiomic insights into *H. difformis* adaptation

The species that shift between C₃ and C₄ pathways reveal regulatory enzyme patterns, subcellular localization, and stress responses critical for optimizing carbon assimilation [6]. In the current study, proteomic analysis of *H. difformis* identified 4900 proteins, with 212 upregulated and 329 downregulated under submergence, showing significant molecular adjustments. This response is more extensive than that in *H. polysperma* (186 differential proteins) [35]. The majority of proteins are localized to the chloroplast (42.7%), cytoplasm (20.15%), and nucleus (14.79%), with minor mitochondrial involvement and some chloroplast–mitochondria dual localization, indicating energy coordination. Heatmaps revealed distinct expression patterns for submerged and terrestrial leaves. Metabolomic profiling identified 537 differentially abundant compounds (156 up, 381 down), mostly affecting flavonoids, terpenoids, and amino acids. The pathways that were enriched included hormone signaling, flavonoid biosynthesis, secondary metabolism, and lipid and amino acid metabolism. The reprogramming scale exceeds that of *Zostera marina* [66], and the patterns are similar to submerged *O. sativa* seedlings [67–69], and the stem of *Salix variegata* [70]. Similar metabolic changes have been observed, soybean [71], *Lemna minor,* and *Salvinia natans* [72], possibly due to ethylene accumulation and CO₂ restriction. Transcriptomic analysis identified thousands of DEGs, the majority are associated with metabolism (43%), stress responses (27%), and organismal systems (13%). These findings are consistent with those reported for *Potamogeton octandrus* (6822 DEGs) [73] and *Isoetes sinensis* (1740 DEGs) [74]. Carbon metabolism, amino acid biosynthesis, and hormone signalling were among the key enriched pathways. A coordinated molecular response to submergence was highlighted by the increased expression of genes associated with ethylene and gibberellin (e.g. GID1, ERF1).

In submerged leaves of *H. difformis*, the marked accumulation of lysophosphatidylcholines, together with shifts in organic acids, amino acid derivatives, small peptides, and nitrogen-rich secondary metabolites, indicates extensive metabolic reprogramming. This complements previously reported anatomical and photosynthetic acclimation in this species. Earlier studies demonstrated that submerged leaves of *H. difformis* exhibit higher underwater photosynthetic rates than terrestrial leaves. This enhancement is attributed to thinner cuticle and cell walls, increased CA activity, and efficient HCO₃^−^ utilization via external CA and bicarbonate transport systems [3, 24]. However, these studies primarily focused on structural and carbon acquisition traits and did not address downstream metabolic adjustments. Our metabolomic data provide this missing layer by revealing coordinated membrane remodeling (lysoPC enrichment), enhanced protein turnover and nitrogen recycling (γ-Glu dipeptides and related peptides), and reprogramming of central carbon metabolism (accumulation of organic acids). These changes likely support energy balance and carbon–nitrogen homeostasis under low dissolved CO₂. Similar metabolic responses have been reported in other aquatic macrophytes, where carbon limitation alters carbon and nitrogen metabolism to sustain growth [75]. Furthermore, studies on submerged plants under environmental stress show reduced carbohydrate reserves but increased amino acid pools, reflecting metabolic reallocation under constrained conditions [76, 77]. The observed increase in alkaloids, phenolamines, terpenoids, and nucleotide-related metabolites further suggests activation of stress-defense pathways and elevated energy demand. Collectively, these findings indicate that submerged adaptation in *H. difformis* involves an integrated response combining anatomical, photosynthetic, and metabolic reprogramming to optimize performance in aquatic environments.

### Multiomic analysis revealed variations in the submerged carbon concentration mechanism

Modified C_4_ photosynthetic pathways, such as C_3_–C_4_ and C_4_-like photosynthesis, have been observed in several plant species, including Eleocharis [78], *Flaveria* [79], *Heliotropium* [80], *Chenopodium* [81], *Ottelia* [7], and *Moricandia* [82]. Some species, such as *Heliotropium* and *Chenopodium*, display a proto-Kranz type of photosynthesis, which is primarily C_3_ but involves the presence of BSCs. These plants have larger BSCs than mesophyll cells, similar to C_4_ species, but their enzyme activities are comparable to those of typical C_3_ plants [80, 81]. In this study, the multiomics investigation of submerged *H. difformis* demonstrated variable control of the C₄ cycle. Notably, α-CA (FC = ∞, *Hdi028772*) and β-CA (FC = 2.33, *Hdi028773*) were significantly upregulated at both the transcript and protein levels, suggesting improved bicarbonate-to-CO_2_ conversion. These results align with previous findings in *H. polysperma* [35] and *H. difformis* [3]. In contrast, PEPC revealed transcript-level downregulation (*Hdi023849, Hdi029117*) but no substantial protein change, indicating decreased carboxylation capability. Similarly, submerged leaves of *H. polysperma* [35] and *Eleocharis vivipara* [83] exhibit decreased C₄ traits in aquatic conditions. However, our results provide a different insight. In contrast to *H. polysperma*, our data indicate that *H. difformis* performs NAD-ME type C₄ photosynthetic characteristics when submerged and C_3_ photosynthesis under terrestrial conditions.

The contrasting regulation of C₄-associated enzymes and the Calvin–Benson cycle in submerged *H. difformis* reveals a distinctive mode of CCM plasticity among aquatic macrophytes. This strategy most closely parallels NAD-ME–type systems described in *Ottelia*, yet differs fundamentally from patterns reported for other heteroblastic or stress-exposed species. In submerged leaves, PEPC and PPDK activities increase sharply despite stable or even reduced protein abundance and clear transcript downregulation. This decoupling between activity, protein level, and transcription indicates that *H. difformis* enhances C₄-like carboxylation predominantly through post-transcriptional and post-translational regulation rather than through bulk enzyme accumulation (Figs 3 and 4). A similar regulatory logic has been documented in *Ottelia alismoides*, where multiomics and enzyme assays demonstrated that a single-cell NAD-ME C₄ + CAM + HCO₃^−^ CCM is implemented via isoform selection, temporal control, and enzyme activation rather than simple increases in protein abundance [25, 56]. Crucially, however, *H. difformis* deploys this logic in a fundamentally different manner. Unlike *O. alismoides*, which sustains high PEPC–PPDK–NAD-ME capacity across CO₂ regimes and operates a broadly upscaled biochemical CCM [25, 56], submerged *H. difformis* combines elevated PEPC and PPDK activities with a strong induction of NAD-ME while simultaneously depleting PPDK protein and its substrate phosphoenolpyruvate.

Our multiomics analysis reveals a coordinated downregulation of the Calvin-Benson cycle in submerged leaves of *H. difformis*. This is characterized by reduced protein levels of RbcL (FC = 0.37), PRK (FC = 0.60), RpiA (FC = 0.58), and GADH (NADP^+^) (FC = 0.37), in addition to lower transcript levels and depleted intermediates (Ru5P, S7P, F6P). This suggests that under submersion, C₃ carbon fixation is significantly inhibited. In mulberry leaves exposed to flooding, key Calvin cycle genes (Rubisco, PRK, ALDO, PGK, GAPDH, TK, and Rpi) were significantly downregulated, resulting in reduced photosynthetic capacity under nonstomatal constraints [84]. Similarly, floating and submerged leaves of *O. ovalifolia* show decreased Rubisco activity, unaffected PEPC, and reduced PPDK activity in submerged leaves, indicating a diminished Calvin cycle [7]. A meta-analysis of C₃ and C₄ plants subjected to drought showed that most carbon fixation genes, including those of the Calvin cycle, were reduced in C₃ species [85]. This adds to the evidence that Calvin cycle inhibition is a frequent response to abiotic stress, including submersion.

The coordinated suppression of Calvin–Benson cycle components in submerged *H. difformis* further emphasizes this strategic shift. Reduced abundance of RbcL, PRK, RpiA, and GADH (NADP^+^), together with depletion of their metabolic intermediates, signals an intentional downscaling of C₃ capacity. This response stands in sharp contrast to submerged *H. polysperma*, where C₄-related proteins decline but Calvin-cycle enzymes are maintained or even reinforced, supporting a transition toward HCO₃-supported C₃ fixation rather than toward a strengthened biochemical CCM [35]. At the same time, selective upregulation of cytosolic aspartate aminotransferase and alanine aminotransferase in *H. difformis*, coupled with stable malate and aspartate pools, suggests that transamination and carbon–nitrogen integration are prioritized over bulk organic acid accumulation. This aligns with studies of submerged macrophytes showing that elevated PEPC and PPDK activities can support ammonium detoxification and tight coupling between carbon and nitrogen metabolism under high NH₄^+^ availability [86].

Figure 8 provides a comprehensive, cross-species comparison of CCMs in *H. difformis*, *O. alismoides*, and *H. verticillata*, integrating transcriptomic, proteomic, and metabolomic data to elucidate their unique adaptations for enhancing CO₂ availability to Rubisco. The comparison underscores the distinct strategies employed by each species in response to fluctuating aquatic environments, highlighting the diversity of C₄-like mechanisms across amphibious plants. In *H. difformis*, the up-regulation of CCM-related genes (*Hdi028772, Hdi028773, Hdi002852*) and proteins (α-CA, β-CA, cAspAT), along with the down-regulation of Calvin cycle enzymes (rbcL, PRK), indicates a shift toward a bicarbonate-driven, single-cell C₄-like metabolism. This streamlined, NAD-ME-biased system reflects a specialized adaptation for submerged conditions, where bicarbonate utilization is crucial for efficient carbon fixation. In contrast, *O. alismoides* exhibits a more flexible and multicomponent CCM strategy, involving the coordinated induction of PEPC, rbcS isoforms, and other key enzymes (PPDK, PEPC, PRK, GAPDH), alongside elevated levels of metabolites such as malate, aspartate, and pyruvate. This integrated system combines aspects of single-cell C₄, CAM, and bicarbonate use, optimizing carbon gain and minimizing photorespiration under low CO₂ conditions [56, 87, 88]. Such flexibility allows *O. alismoides* to thrive in highly fluctuating aquatic environments, showcasing its adaptability in response to diverse environmental stresses. Similarly, *H. verticillata* activates key CCM-related genes (Hvpepc, Hvppdk, Hvme, PPDK) and metabolites (malate, PEP, OAA) to enhance bicarbonate assimilation under stress [13, 21, 89–92]. While similar to *H. difformis* in terms of bicarbonate utilization, *H. verticillata* employs a more complex, multipathway strategy, reflecting a higher degree of metabolic flexibility in response to variable environmental conditions.

**Figure 8:**
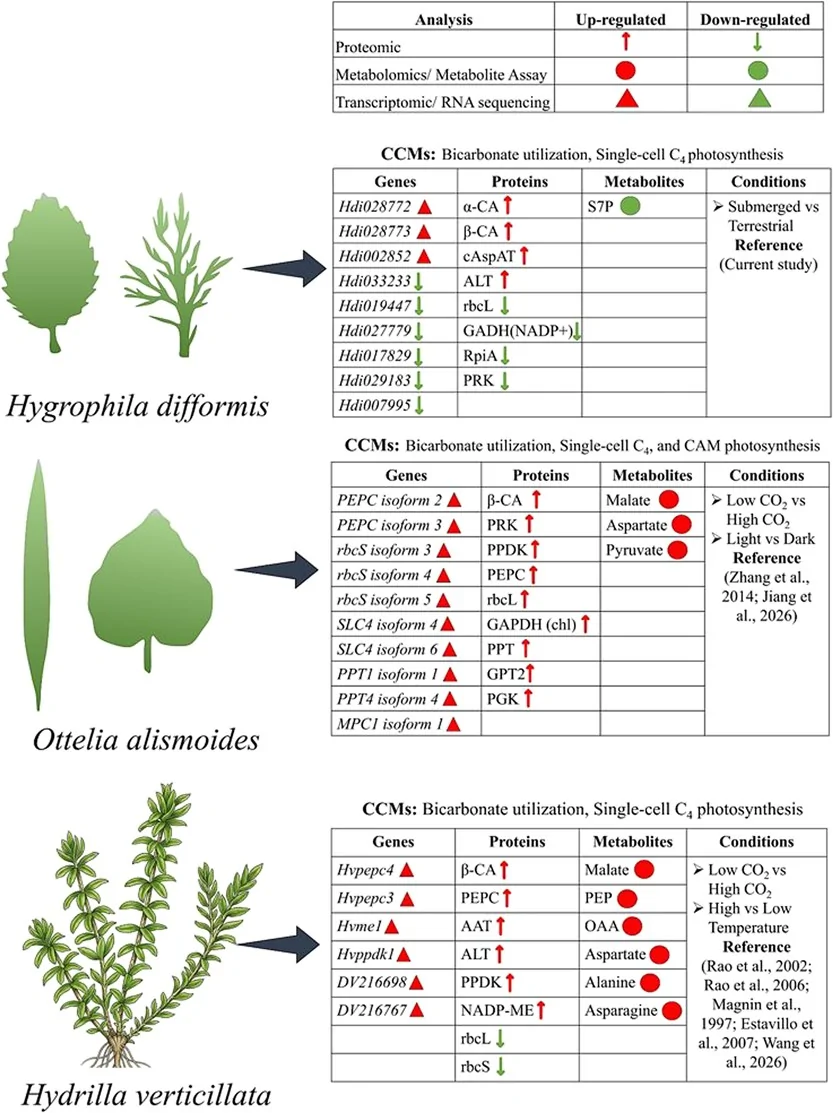
Comparative multiomics analysis of bicarbonate-based carbon concentrating mechanisms in aquatic plants. Transcriptomic, proteomic, and metabolomic data illustrate the regulation of genes, proteins, and metabolites associated with bicarbonate utilization and alternative photosynthetic pathways (single-cell C₄ and CAM) in *H. difformis*, *O. alismoides*, and *H. verticillata* under different environmental conditions.

In summary, while *H. difformis* utilizes a streamlined, NAD-ME-biased C₄-like system that is optimized for submerged conditions, *O. alismoides* and *H. verticillata* adopt more flexible, multicomponent CCM strategies that reflect their unique adaptations to fluctuating aquatic environments. This comparative analysis highlights the distinct, species-specific adaptations of each plant, emphasizing the evolutionary diversity in amphibious plants’ responses to CO₂ availability and environmental stresses.

### Carbonic anhydrases may function in the submerged adaptation of *H. difformis*

CAs are widely distributed enzymes that participate in a variety of metabolic activities. The function of CAs in several physiological processes in photosynthetic organisms has been the subject of extensive research over the past few decades [3, 93–98]. Advances in whole-genome sequencing have allowed the genome-wide identification of *CA* gene families in several species [3, 99, 100, 101]. Three types of CAs (α-type, β-type, and γ-type) have been found in higher plants, each has several functional isoforms [102–104]. Previously, Horiguchi *et al*. [3] performed the transcriptome analysis of *H. difformis* based on *de novo* assembly using Trinity. They revealed 17 CAs, including 7 α-CAs, 6 β-CAs, and 4 γ-CAs. The expression levels of putative α- CA and β-CA genes increased significantly in the submerged leaves compared to the terrestrial leaves *H. difformis*, including two upregulated α-CAs, one upregulated β-CA, three downregulated α-CAs, and three upregulated β-CAs. We recently published the genome data of *H. difformis* [105], making it possible to re-identify CAs and analyze their expression in this species.

In this study, we identified a total of 21 CA members in *H. difformis*, including 14 α-CA, 5 β-CA, and 2 γ-CA. The data indicate that the most prevalent kind of CAs are α-type, indicating their functional significance and evolutionary conservation. Furthermore, we found segmental duplications between five *HdCA* pairs and analyzed the synteny of *CAs* between diverse species. The distribution patterns emphasize gene duplication occurrences and are consistent with previous studies on CA conservation [104, 106, 107]. The heatmap analysis of *cis*-regulatory elements (CREs) in CA genes revealed a variety of regulatory patterns related to phytohormone responses and abiotic stress tolerance. Certain CREs, such as ABRE and TCA elements, imply functions in the regulation of abscisic acid and stress responses, respectively. Essential functions of cis-regulatory elements include coordinating reactions to environmental and developmental signals [108–110]. Therefore, CAs in *H. difformis* may respond to various environmental factors and phytohormones through these *cis*-elements.

Horiguchi et al. [3] reported variations in *CA* gene expression between terrestrial and submerged leaves of *H. difformis*. However, their transcriptome analysis relied on a single sample from each condition, with transcripts predicted through de novo assembly. This approach compromises the reliability of their findings and may lead to inaccuracies in the future. In the present study, we integrated phenotypic, anatomical, and physiological analysis alongside transcriptome profiling Our results found that *HdαCA9* exhibited a remarkable 194-fold increase under submergence, indicating that it may play a crucial role in adaptation to submerged conditions.

*CA* genes are involved in a variety of metabolic events, including photosynthesis, respiration, pH control, inorganic carbon transport, ion transport, and water and electrolyte balance [104, 111–113]. To further understand their involvement in growth, development, and photosynthesis, future research ought to develop transgenic plants that overexpress *HdCAs* or *HdCA*-knockout mutants. Additionally, protein interaction networks should be used to further investigate their biological activities in other metabolic processes.

### Submerged leaves exhibit heterogeneous chloroplasts with limited starch accumulation

Our integrated multiomics and ultrastructural analysis demonstrate that *H. difformis* significantly suppresses starch bioproduction and modifies starch degradation in submerged conditions. The transcript levels of essential starch biosynthetic enzymes (AGPase, GBSS, SS, SBE, and DBE) were significantly decreased, with GBSS and DBE also reduced at the proteomic level. This is consistent with observations in rice, where submergence reduced AGPase and GBSS, affecting starch accumulation and mobilization during anaerobic germination [114–116]. Similarly, the starch-deficient Arabidopsis *pgm* mutant shows reduced survival under submergence, highlighting the importance of starch reserves in early stress adaptation [117]. Recently, Yang *et al*. [118] reported that Arabidopsis plants exhibited a marked reduction in starch granule accumulation in chloroplasts under submerged conditions compared to those grown terrestrially, reflecting metabolic adjustments to low oxygen levels. In *H. difformis*, transcripts encoding α-amylase were upregulated while protein abundance was reduced, indicating post-transcriptional modulation. This is consistent with patterns observed in deep-water rice internodes, which exhibit a 70-fold increase in amylolytic activity and 65% starch depletion during three days of submergence [119]. The difference between transcript and protein levels is most likely due to the regulatory mechanisms that regulate starch breakdown in response to actual metabolic demand. Metabolomics indicated a significant reduction in glucose content, which is consistent with a general inhibition of starch metabolic flux. Similar results have been observed in aquatic systems, such as *Alternanthera philoxeroides*, where starch content continuously decreases after prolonged submergence at a range of temperatures [120]. Similarly, submerged *Ottelia alismoides* alters starch production in response to acidity flow, indicating altered carbohydrate metabolism under aquatic conditions [88].

The ultra-structural analysis of submerged leaves of *H. difformis* revealed the presence of dimorphic chloroplasts within the mesophyll cells. While some chloroplasts were small and lacked starch granules, others contained well-developed starch deposits. This variation suggests a possible functional specialization among chloroplast types within the same cell. It has been reported previously that the species from the Hydrocharitaceae family exhibit C_4_ photosynthesis despite lacking Kranz anatomy [12, 17, 54, 56, 59]. Similarly, *H. verticillate* and *Ottelia alismoides* perform single-cell C_4_ photosynthesis, resembling mechanisms seen in terrestrial species such as *Bienertia cycloptera* and *Suaeda aralocaspica,* where carbon fixation and decarboxylation are spatially separated within one cell [11, 121–123]. The dimorphic chloroplasts, structurally different chloroplasts within a single cell, are reported in both terrestrial and aquatic species [123, 124]. In *Cabomba caroliniana,* mesophyll chloroplasts are larger, with more developed grana and greater starch accumulation than those in epidermal cells [125]. Similarly, members of Posdostemaceae have small and starchless chloroplasts in the upper epidermis, while the mesophyll contains larger chloroplasts with more thylakoids and abundant starch granules [126].

### Evolutionary origin and adaptive significance of C_4_-like pathways in *H. difformis* and comparative physiology of amphibious plants

Amphibious plants exhibit remarkable photosynthetic plasticity, adjusting their carbon acquisition strategies when transitioning between aerial and submerged conditions. For instance, in *E. vivipara*, culms utilize C_4_ photosynthesis with Kranz anatomy on land, but switch to C_3_ photosynthesis when submerged, as confirmed by δ^13^C, enzyme activity, and ^14^C-labeling studies [127]. Other *Eleocharis* species display a gradient of C_4_ to C_3_–C_4_ intermediates and even strong bicarbonate utilization underwater, suggesting physiological adaptation to low CO₂ conditions [78, 128]. Similarly, submerged leaves of amphibious macrophytes often show reductions in stomata and Rubisco, accompanied by altered PEPC activity and enhanced O₂ evolution compared to strictly C_3_ relatives, which further indicate specialization for submerged environments with high diffusion resistance [2]. *Ottelia alismoides* employs a combination of C_4_ and CAM strategies, utilizing single cells to integrate multiple CCMs without the need for Kranz anatomy [87]. These adaptations help them cope with fluctuating CO₂ and light conditions in aquatic habitats, a trait also observed in other species such as *H. verticillata* and *E. densa*, where facultative C_4_ photosynthesis is deployed depending on environmental cues [62].

Comparative genomic studies have revealed how photosynthetic plasticity is genetically enabled in amphibious plants. In *E. vivipara*, genome-scale analyses have identified a recent whole-genome duplication, extensive chromosomal rearrangements, and subgenome-specific expression of C_4_-related enzymes in terrestrial organs, with C_3_ metabolism used underwater [129, 130]. Similarly, transcriptomic studies in *H. verticillata* highlight the broad regulatory reprogramming required for the induction of C_4_-like systems, with hundreds of genes, including C_4_ enzymes and transcription factors, showing differential expression between C_3_ and C_4_ states [90, 131]. Comparative transcriptomics across angiosperms suggests that many gene lineages can be co-opted into C_4_ photosynthesis, often involving genes with pre-existing favorable expression patterns, which are then restructured during the evolution of C_4_-like systems [132].

The evolution of C_4_-like systems in aquatic environments follows a consistent pattern of co-opting existing biochemical components to enhance carbon fixation when CO₂ is limited. This pattern is evident in species such as *Hydrilla* [21], *Orcuttia* [133], and *H. difformis*, where the C_4_-like pathways emerge as adaptations to low CO₂ availability and fluctuating carbon levels in aquatic systems. The challenge in aquatic environments is not only the limited availability of CO₂, which diffuses much slower in water compared to air, but also the diel fluctuations in CO₂, which make carbon fixation even more challenging. C_4_-like pathways help overcome these challenges by concentrating CO₂ around Rubisco, optimizing carbon fixation even in CO₂-depleted conditions [134].

Our findings in *H. difformis* indicate that under submerged conditions, the species transitions to a C_4_-like pathway, enhancing its carbon gain efficiency in low-CO₂ environments. This supports the view that C_4_-like mechanisms in aquatic plants are essential not just for mitigating photorespiration but also for optimizing carbon fixation in the face of insufficient atmospheric or aquatic CO₂. These pathways also provide distinct advantages in environments where CO₂ fluctuates significantly between day and night, a feature common in submerged ecosystems. Additionally, the co-occurrence of C_4_ and CAM pathways in some aquatic plants further highlights the adaptive strategies in these environments. This strategy is particularly beneficial in oligotrophic or low-CO₂ environments, where the availability of inorganic carbon is limited.

The evolution of C_4_-like pathways in *H. difformis* aligns with broader trends observed in other amphibious and aquatic species. Through biochemical and genetic shifts, rather than major anatomical changes, these plants have developed effective carbon-concentrating mechanisms to cope with the challenges posed by low CO₂ availability and fluctuating environmental conditions. These adaptations allow species like *H. difformis* to thrive in submerged environments where CO₂ and light availability are variable, and they illustrate how regulatory shifts and the co-option of existing enzymatic functions can drive the evolution of complex metabolic strategies such as C_4_-like photosynthesis.

## Conclusion

This study provides a comprehensive and integrative view of the morphological, anatomical, physiological, and molecular adaptations that enable *H. difformis* to survive and perform photosynthesis under submerged conditions. Submerged leaves are finely dissected, exhibit an increased surface area, and display enhanced HCO₃^−^ utilization, traits that are well-suited to the low-CO₂ aquatic environment. Although classical Kranz anatomy is absent, the elevated activities of PPDK, PEPC, and NAD-ME, together with the upregulation of key enzymes (e.g. cAspAT, ALT), provide compelling evidence for the induction of an NAD-ME–type C₄-like photosynthetic program. These findings reveal a previously unrecognized carbon fixation strategy operating in submerged leaves of *H. difformis*. Multiomics analyses further demonstrate that submergence triggers extensive molecular reprogramming in *H. difformis*. Carbon metabolism and stress-related pathways are strongly reshaped, reflecting a coordinated acclimation to the underwater environment. Core enzymes of the C₃ cycle are markedly downregulated, while CA is strongly upregulated, enhancing CO₂ acquisition from HCO₃^−^ and reinforcing the transition toward C₄-like biochemical function. These results highlight the exceptional phenotypic and metabolic plasticity of *H. difformis*, which allows it to optimize carbon acquisition, energy use, and growth across sharply contrasting terrestrial and aquatic habitats. By combining heterophylly with flexible deployment of NAD-ME–biased C₄-associated biochemistry and bicarbonate-based carbon uptake, *H. difformis* occupies a unique position along the continuum of photosynthetic strategies in amphibious plants.

### Future prospects

This study establishes a framework for understanding carbon-concentrating mechanisms (CCMs) in *H. difformis*, but several aspects require further investigation. While transcriptional regulation is evident, discrepancies between enzyme activity and gene/protein levels suggest an important role for posttranscriptional and posttranslational regulation, which should be explored using proteomics and PTM-focused analyses. In addition, functional validation of key genes (e.g. HdαCA9, PEPC, NAD-ME) and isoform-specific characterization of CA family members will be essential to confirm their roles in HCO₃^−^ utilization and carbon fixation.

Furthermore, although metabolite changes were linked to candidate genes, the underlying regulatory networks and metabolic fluxes remain to be resolved. Future studies should also investigate the role of starch metabolism in CCM function, incorporate time-resolved experiments under varying environmental conditions, and perform comparative analyses across species. Such efforts will help translate these findings toward improving carbon use efficiency and stress adaptation in economically important crops.

## Methodology

### Plant materials and growing conditions

Plants were obtained from the Institute of Hydrobiology, Chinese Academy of Sciences, Hubei Province. Plantlets were propagated vegetatively, potted in soil, and maintained in growth chambers under regulated conditions, including 16 hours of light daily, 23°C temperature, and 60 μmol/m^2^/s LED white light flux. Terrestrial plantlets were watered once every seven days, with a relative humidity (RH) of 30%. After 30 days, terrestrial and submerged leaves were harvested for morphological, anatomical, physiological, and multiomic analysis.

### Morphological and anatomical observations

Leaf morphology was determined by measuring fresh weight, dried weight, area, length, width, leaf mass per area (LMA), and dissection index (DI). The dissection index of the leaves was calculated by using the following formula:


$$ DI=\frac{\mathrm{Leaf}\ \mathrm{perimeter}}{\sqrt{\mathrm{Leaf}\ \mathrm{area}}} $$

Fresh leaves were photographed using a Cannon D60 Professional camera. The length, width, and area of leaves were measured with Image-J software 1.50 (NIH, USA).

For scanning electron microscopy (SEM), leaf portions were vacuum-infiltrated with 2.5% glutaraldehyde in 0.1 M phosphate buffer (pH 7.4) and fixed overnight at 4°C. The samples were then washed in the buffer and dehydrated using an ethanol series, followed by critical point drying with liquid CO_2_. The specimens were placed on stubs and coated with gold using a sputter coater before being examined with a Hitachi S-4800 scanning electron microscope. Each treatment has three replicates. After 1.5 months, mature leaves were collected and examined for morphological changes.

Leaves were segmented (3 × 3 mm) and fixed overnight in 2.5% glutaraldehyde in 0.1 M phosphate buffer (pH 7.4) at 4°C, followed by 2.5 hours of postfixation in 1% OsO_4_ at 4°C [11]. The samples were dehydrated using a graded ethanol series (30%, 50%, 70%, 90%, and 100%) and then processed in an ethanol–acetone solution. They were then immersed in a 1:1 combination of acetone and epoxy resin for 1 hour, followed by 1:2 for 8 hours, before being implanted in SPI-PON 812 for 48 hours at 60°C. The Leica EM UC7 ultramicrotome was used to obtain ultrathin sections (72 nm). The ultrathin sections were examined and photographed with an HT7700 transmission electron microscope (Hitachi High-Tech, Tokyo, Japan) after being stained with uranyl acetate and lead citrate.

### Photosynthetic pigments

The technique established by Kumar *et al*. [135] was adopted with slight modifications to determine chlorophyll and carotenoid concentrations in *H. difformis* leaves [135]. One hundred milligrams of fresh leaves were homogenized with 95% ethanol and centrifuged at 7000 × *g* for 10 minutes. The supernatant was collected, and the absorbance (*A*) was measured using an ELISA plate reader (i3x Molecular Devices, United States) at 663 nm for Chlorophyll-A, 646 nm for Chlorophyll-B, and 470 nm for carotenoids. The following formulae were used to compute the concentrations of chlorophyll and carotenoids:

Chlorophyll-A = 12.21 × *A*_663_ − 2.81 × *A*_646_.

Chlorophyll-B = 20.13 × *A*_646_ − 5.03 × *A*_663_.

Total chlorophyll = Chl-a + Chl-b.

Carotenoids = [1000 × *A*_470_ − 3.27 × (Chl-a) − 104(Chl-b)]/229.

### pH-drift experiments

pH drift experiments were performed to assess HCO₃^−^ consumption capacity in *H. difformis*. Both terrestrial and submerged leaves were analyzed using three biological replicates. Following leaf area determination, intact leaves were incubated in a solution containing 10 mM NaHCO₃, 1.5 mM KCl, and 1 mM NaCl for 24 hours at 23°C under 60 μmol m^−2^ s^−1^ white light. After incubation, the pH of the medium was measured using a pH meter (LAQUA F-51, Horiba, Kyoto, Japan), with incubation medium lacking leaves used as a blank control. The HCO_3_^−^ utilization ability of terrestrial and submerged leaves of *H. difformis* was determined by using the following formula:

HCO_3_^−^ Utilization Ability = (pH_sample_ − pH_blank_)/Leaf area.

Photosynthetic rates of plants adapted to various carbon sources were determined at pH 6.3 and 8.3. To optimize CO_2_ or HCO_3_^−^ utilization, the medium pH was changed to 6.3 or 8.3 using 1 M HCl or NaOH. Acetazolamide (AZ) and ethoxyzolamide (EZ) stock solutions were prepared at a concentration of 10 mM by dissolving them in 50 mM NaOH for the inhibitor experiments. AZ inhibits exterior CA activity, whereas EZ targets internal CA activity. In addition, solutions of 4,4′-diisothiocyanatostilbene-2,2′-disulfonate (DIDS), an inhibitor of anion exchange proteins, and Tris (hydroxymethyl) aminomethane (TRIS), which inhibits the HCO_3_^−^/H+ symport, were also synthesized. Following that, the photosynthetic oxygen evolution rate was assessed in the presence of 50 mM TRIS, 0.3 mM DIDS, 0.1 mM EZ, and 0.1 mM AZ under HCO_3_^−^ conditions (pH 8.3).

### Measurement of the stable 13C isotope

Initially, both submerged and terrestrial leaves were oven-dried. The stable 13C isotope concentration was then determined using a Delta Plus Advantage isotope ratio mass spectrometer (Thermo Fisher Scientific, Bremen, Germany) [18, 136]. Isotope data is provided in the conventional delta (δ) notation (‰) relative to the Vienna Pee Dee Belemnite (V-PDB) standard.

### Measurement of photosynthesis enzyme activities

Enzyme activities in terrestrial and submerged leaves of *H. difformis* were quantified to assess key metabolic processes associated with carbon fixation and carbon utilization. The activities of ribulose-1,5-bisphosphate carboxylase/oxygenase, phosphoenolpyruvate carboxylase, pyruvate phosphate dikinase, NADP-malic enzyme, NAD-malic enzyme, and CA were determined using commercially available assay kits, and all procedures were performed in accordance with the manufacturers’ protocols.

Terrestrial and submerged leaves were harvested from plants grown under controlled experimental conditions. Immediately after collection, leaf samples were placed on ice to limit enzymatic degradation and transported for processing. For each assay, 0.1 g of fresh leaf tissue was homogenized in 1 ml of the appropriate extraction buffer supplied with the corresponding assay kit. The homogenates were centrifuged at 10 000 × *g* for 10 minutes at 4°C, and the resulting supernatants were carefully collected and kept on ice for immediate enzymatic analysis.

Rubisco activity was determined using the BC0440 assay kit (Solarbio, Beijing, China), which quantifies enzyme activity by monitoring the decrease in NADH absorbance at 340 nm. Absorbance values were recorded before and after incubation for 5 minutes at 25°C using a spectrophotometer. Phosphoenolpyruvate carboxylase activity was measured using the BC2190 kit (Solarbio, Beijing, China), based on the consumption of NADH in a coupled reaction system, with absorbance measured at 340 nm before and after incubation for 5 minutes at 30°C. Pyruvate phosphate dikinase activity was assessed using the BC5850 kit (Solarbio, Beijing, China) by monitoring changes in NADH absorbance at 340 nm following incubation for 5 minutes at 37°C.

NADP-malic enzyme activity was determined using the BC1120 kit (Solarbio, Beijing, China), which measures NADPH formation as an increase in absorbance at 340 nm. Absorbance was recorded before and after incubation for 1 minute at either 25°C or 37°C, depending on the experimental conditions. NAD-malic enzyme activity was measured using the BC1130 kit (Solarbio, Beijing, China) by detecting NADH production at 340 nm following incubation for 1 minute at 25°C or 37°C. CA activity was analyzed using the BC5440 kit (Solarbio, Beijing, China), a colorimetric assay based on p-nitrophenol formation, with absorbance measured at 405 nm before and after incubation for 5 minutes at 37°C. A standard solution supplied with the kit was used for reference measurements.

All absorbance measurements were performed using a spectrophotometer equipped with 1 ml quartz cuvettes for readings at 340 nm and 1 ml glass cuvettes for readings at 405 nm. All reagents required for enzymatic analyses were provided within the respective commercial assay kits. Enzyme activities were calculated according to the manufacturer’s instructions and expressed as units per milligram of protein.

### Sampling for multiomics

Plantlets were vegetatively propagated and grown under controlled environmental conditions (16 h photoperiod, 23°C, and 60 μmol m^−2^ s^−1^ white LED light). Terrestrial plants were maintained under aerial conditions, while submerged plants were fully acclimated to aquatic conditions. After 30 days, fully expanded leaves (third from the apex) were collected from both growth forms. For omics analyses, three independent biological replicates were used for each condition.

### Proteomic analysis

Protein extraction and analysis were performed by Metware Biotech (Wuhan, China) to investigate the proteome differences between submerged and terrestrial leaves of *H. difformis* grown at a constant temperature of 23°C. Leaf samples from both conditions were processed in triplicate to ensure biological repeatability. Following centrifugation, four volumes of cooled acetone were used to precipitate the proteins that were present in the supernatant.

The precipitated protein pellets were dissolved in 8 M urea and then reconstituted with 100 mM Tris-HCl buffer. Protein was reduced with 10 mM dithiothreitol (DTT) at 37°C for 1 hour, then alkylated with 40 mM iodoacetamide at 25°C in the dark for 30 minutes. Protein concentration was determined using the Bradford assay. The urea content was reduced to <2 M by dilution with 100 mM Tris–HCl (pH 8.0). Proteins were digested with trypsin at a 1:50 enzyme-to-protein ratio (w/w) and incubated overnight at 37°C. The digestive reaction was terminated by adjusting the pH to 6.0 using trifluoroacetic acid.

After centrifugation at 12 000 *g* for 15 minutes, peptides were purified with a Sep-Pak C18 desalting column, vacuum-dried, and kept at −20°C until further analysis. Peptides were examined for proteome profiling using liquid chromatography–tandem mass spectrometry (LC-MS/MS) using an Orbitrap Exploris 480 mass spectrometer and an Easy-nLC 1200 system. Raw MS data were processed with the library-free technique in DIA-NN v1.8 software, which used deep-learning algorithms to predict spectral libraries from MS data. The match-between-runs (MBR) function was used to generate a spectrum library from data-independent acquisition (DIA) data, which was then utilized for reanalysis. Protein identification was carried out using a 1% false discovery rate threshold at both the precursor and protein levels. Protein expression analysis was performed using DIA-NN output files. Significantly changed proteins were detected with an FC threshold of >1.3 and a *P*-value <.05.

### Metabolomic analysis

Plant samples were freeze-dried and mashed to a fine powder before being extracted with a 70% methanolic aqueous internal standard solution at −20°C. The mixture was vortexed, centrifuged, and filtered through a microporous membrane; the resultant supernatant was preserved for UPLC-MS/MS analysis. The extracts were examined using an ExionLC™ AD UPLC and a tandem mass spectrometry (MS/MS) system. The UPLC conditions featured a gradient program that began with 95% solvent A (water with 0.1% formic acid) and 5% solvent B (acetonitrile with 0.1% formic acid), progressed to 5% A and 95% B, and then returned to the initial conditions. The flow rate was 0.35 ml/min, with a column temperature set to 40°C and an injection volume of 2 μl. The effluent was fed into an ESI-triple quadrupole-linear ion trap (QTRAP)-MS.

The data were analyzed using unsupervised PCA (principal component analysis) in R with unit variance scaling. Heatmaps were used to show hierarchical cluster analysis and Pearson correlation coefficients. VIP scores (VIP > 1) and absolute Log_2_FC (|Log_2_FC| ≥ 1.0) were used to identify differential metabolites, which were then validated with OPLS-DA and permutation testing. The identified metabolites were annotated using the KEGG database and assigned to KEGG pathways for enrichment analysis.

### Transcriptomic analysis

RNA was extracted from plant samples using ethanol precipitation and CTAB-PBIOZOL, followed by dissolution in diethyl pyrocarbonate (DEPC) treated water. RNA quality and concentration were measured with a Qubit fluorescence quantifier and a Qsep400 biofragment analyzer. PolyA-tailed mRNAs were enriched with Oligo (dT) magnetic beads, then fragmented and reverse-transcribed into strand-specific cDNAs. Following library amplification and concentration, and fragment size verification, the libraries were sequenced using an Illumina platform to obtain 150 bp pair-end reads.

Fastp was used to remove low-quality reads and adapters from the data. Clean readings were aligned to the reference genome with HISAT, and new transcripts were predicted using StringTie. Gene expression levels were measured using featureCounts, and fragments per kilobase million (FPKM) values were calculated. DESeq2 was used to evaluate differential expression, with *P*-values corrected using Benjamini–Hochberg. Pathway enrichment was determined using KEGG and GO terms, and alternative splicing events were identified using rMATS. SNPs were identified using GATK and annotated with Annovar. The STRING database was used to examine protein–protein interactions, which were then further studied using Gene Set Enrichment Analysis and Weighted Gene Co-expression Network Analysis with default parameters.

### Identification of carbonic anhydrases

The genome and protein data of *H. difformis* were downloaded from GenBank (https://www.ncbi.nlm.nih.gov/genbank/, accession number: PRJNA872356). All the CA protein sequences of *A. thaliana* were downloaded from the NCBI website and used as query sequences to search for CA in *H. difformis* by BLASTp with an E-value cutoff set as 1e^− 5^. Simultaneously, the HMM profiles of the Carb_anhydrase (PF00194), Pro_CA (PF00484), and Hexapep (PF00132) domains were obtained from the Pfam database (http://pfam.xfam.org/), and hmmSearch tools in the HMMER server under default parameters were used to identify CA proteins of *H. difformis* (Table S3). Putative CAs were further confirmed by the SMART database (http://smart.embl-heidelberg.de) and NCBI Conserved Domain database (http://www.ncbi.nlm.nih.gov/Structure/cdd/wrpsb.cgi).

### Phylogenetic classification

Neighbor-joining phylogenetic tree of all identified CAs was constructed in MEGA 7.0 (http://www.megasoftware.net/) with 1000 bootstrap replicates on the JTT model and displayed using the online software iTOL (https://itol.embl.de).

### *Cis*-acting elements, chromosomal localization, gene duplication, and Synteny analysis

The 2.0 kb promoter sequence of all *CA* genes was extracted by TBtools software. The *cis*-acting elements of the promoter sequence were analyzed by PLANTCARE (http://bioinformatics.psb.ugent.be/webtools/plantcare/html/) and visualized by TBtools. The chromosomal distribution of *CA* genes was determined from the annotation file and visualized by TBtools. The synteny and gene duplication of *CA* genes were analyzed by MCScanX and TBtools. The syntenic relationships among *H. difformis* and other representative species were also identified by MCScanX and visualized by TBtools [137].

### Reverse-transcription quantitative PCR

Total RNA was extracted from mature leaves of terrestrial or submerged plants maintained in growth chambers under regulated conditions, including 16 hours of light daily, 23°C temperature, and 60 μmol/m^2^/s white light flux. Terrestrial plantlets were watered once every 7 days, with a 30% RH. RNA extractions were then used to synthesize cDNA, as described previously [138]. RT-qPCR was performed using the SYBR Premix Ex Taq Kit (Takara) in a CFX96 Real-Time PCR system (Bio-Rad). Experiments were performed in triplicate from three independent tissue RNA extractions. *HdACTIN1* (*HdACT1*) was used as an internal reference. The 2^−ΔΔCt^ method was employed to calculate the genes’ relative expression. The relative expression of each gene was normalized to terrestrial mature leaves. Primer information is given in Table S2.

### Statistical analysis

All results are shown as means ± standard deviation of three replicates for each treatment. A one-way analysis of variance was used to determine statistical significance (*P* ≤ .05) between the variables under consideration. For multiple comparisons, JMP statistical software v9.0.2 (SAS Institute, NC, United States) was used to run one-way ANOVA followed by Tukey’s honestly significant difference test.

## Supplementary Material

Web_Material_uhag230

## Data Availability

Data are available in the supplementary material.
