# Supplementary material for: Physiological and multiomics analyses on photosynthesis and carbon utilization reveal biochemical carbon-concentrating mechanisms in Hygrophilla difformis under submergence
Source: Hortic Res. 2026 Jun 15;13(11):uhag230. doi: 10.1093/hr/uhag230 (PMC13722866; doi:10.1093/hr/uhag230)
Supplement: Supplementary file 1 [file web_material_uhag230.docx]

*Research Article*

**Physiological and Multi-Omics Analyses on Photosynthesis and Carbon Utilization Reveal Biochemical Carbon Concentrating Mechanisms (CCMs) in *Hygrophilla difformis* Under Submergence**

Abeer Kazmi^1,2 †^, Gaojie Li^1,2 †^, Jingjing Yang^1, 2^, Xuyao Zhao^1, 2^, Md. Fakhrul Islam^1, 2^, Xiaozhe Li^1, 2^, Seisuke Kimura^3, 4^, Keiko U. Torii^5,6,7^, Hongwei Hou^1,2, *^

^1^*The State Key Laboratory of Freshwater Ecology and Biotechnology,* *Institute of Hydrobiology, Chinese Academy of Sciences, Wuhan 430072, Hubei, PR China*

^2^*University of Chinese Academy of Sciences, Beijing 100049, PR China*

^3^*Department of Industrial Life Sciences, Faculty of Life Sciences, Kyoto Sangyo University, Kyoto, Japan*

^4^*Center for Plant Sciences, Kyoto Sangyo University, Kyoto, Japan*

^5^*Institute of Transformative Bio-Molecules (WPI-ITbM), Nagoya University, Nagoya, Aichi, Japan.*

^6^*Howard Hughes Medical Institute, The University of Texas at Austin, Austin, TX, USA.*

^7^*Department of Molecular Biosciences, The University of Texas at Austin, Austin, TX, USA*

***Corresponding Author:** Email: [houhw@ihb.ac.cn](mailto:houhw@ihb.ac.cn)

^†^**Equal Contribution:** Abeer Kazmi and Gaojie Li

**Supplementary Figure 1:** Proteomic analysis of submerged vs. terrestrial leaves of Hygrophila difformis. (A) Volcano plot showing differentially expressed proteins (DEPs), where red and green dots represent significantly upregulated and downregulated proteins, respectively. (B) Pie chart illustrating the subcellular localization of identified proteins, with the majority localized in the chloroplast, cytoplasm, and nucleus. (C) Heatmap of hierarchical clustering based on protein expression profiles in terrestrial (T23a, T23b, T23c) and submerged (S23a, S23b, S23c) conditions, showing distinct proteomic differences between the two environments.

**Supplementary Figure 2:** KEGG pathway classification and differential expression of proteins in submerged and terrestrial Hygrophila difformis leaves. (A) The KEGG pathway classification of differentially expressed proteins (DEPs), categorized into metabolism, cellular processes, and environmental/genetic information processing. (B) The number of upregulated (red) and downregulated (blue) proteins in each KEGG pathway, highlighting key metabolic and regulatory changes in response to submergence.

**Supplementary Figure 3:** Metabolomic profile comparison of submerged and terrestrial leaves of H. difformis. (A) Volcano plot showing the differentially expressed metabolites between leaf types. Green and red dots represent upregulated and downregulated metabolites, respectively. (B) OPLS-DA plot illustrating the distinct clustering of submerged and terrestrial leaves based on metabolomic data. (C) Heatmap showing the distribution of metabolite classes and their relative abundance across the two leaf groups.

**Supplementary Figure 4:** KEGG pathway analysis and differential abundance of metabolites in submerged vs. terrestrial leaves of H. difformis. (A) KEGG pathway classification of metabolites, with emphasis on major metabolic processes like flavonoid biosynthesis and amino acid metabolism. (B) Differential abundance analysis showing metabolic shifts in pathways such as flavonoid biosynthesis, photosynthesis, and lipid metabolism.

**Supplementary Figure 5:** Transcriptomic differences between submerged and terrestrial leaves of Hygrophila difformis. (A) Volcano plot showing differentially expressed genes (DEGs), with 3,749 genes upregulated and 4,032 downregulated in submerged leaves. (B) PCA plot illustrating clear separation between submerged (S) and terrestrial (T) groups based on gene expression profiles. (C) Heatmap of Z-score normalized gene expression, highlighting distinct clustering and expression patterns between the two conditions.

**Supplementary Figure 6:** KEGG pathway enrichment analysis of differentially expressed genes (DEGs) in Hygrophila difformis under submerged versus terrestrial conditions. (A) Bar graph showing the top enriched KEGG pathways grouped by functional categories. Metabolic pathways dominate the DEGs, especially those involved in secondary metabolite biosynthesis, starch and sucrose metabolism, and amino acid metabolism. (B) Circular plot illustrating the distribution of upregulated (red) and downregulated (blue) genes across major KEGG categories. Each ring represents a pathway, with bar height indicating gene count and color scale reflecting statistical significance (–log10 Q-value). Metabolic and environmental information processing pathways show strong differential expression under submergence.

**Supplementary Figure 7:** Starch metabolism in *H. difformis* under submerged and terrestrial conditions. (A) Multiomic analysis of starch synthesis and degradation enzymes, showing fold change (FC) in protein expression. (B) TEM of a chloroplast of a terrestrial leaf. (C) TEM of a submerged leaf cell displaying two distinct chloroplast types. (D) Starch-lacking chloroplast present in the submerged leaves. (E) Chloroplast with starch granules present in submerged leaves. Chl= chloroplast, Sta= starch

**Supplementary Figure 8:** Phylogenetic tree of carbonic anhydrase (CA) isoforms across three distinct classes (α-type, β-type, γ-type) from Arabidopsis thaliana (At) and H. difformis (Hd). The tree highlights the evolutionary relationships between the CA isoforms, with bootstrap values shown at the nodes. Red circles represent H. difformis CAs, and black circles represent Arabidopsis thaliana CAs.

**Table S1.** Highly upregulated metabolites in submerged leaves of *Hygrophila difformis* showing metabolic reprogramming under aquatic conditions

| **Growth Condition** | **Metabolite** | **Class II** | **Log2FC** | **KEGG Pathway** |
| --- | --- | --- | --- | --- |
| Submerged vs Terrestrial Leaves of *Hygrophila difformis* | LysoPC 22:6 | Lipids | 9.454558 | - |
|  | 9-pentofuranuronosyl-9h-purin-6-amine | Nucleotides and derivatives | 8.877237 | - |
|  | γ-Glu-Leu | Amino acids and derivatives | 8.365436 | - |
|  | Ile-Asn | Amino acids and derivatives | 7.077705 | - |
|  | Dihydrocaffeoyltyramine | Phenolamine | 7.048388 | - |
|  | 5-Aminocycloheptane-1,2,3-triol | Alkaloids | 6.992827 | - |
|  | Phenethylamine | Benzylphenylethylamine alkaloids | 6.891763 | Phenylalanine metabolism, Metabolic pathways |
|  | 13-Carboxy-blumenol C | Monoterpenoids | 6.767083 | - |
|  | Hordenine | Alkaloids | 6.560228 | Tyrosine metabolism, Metabolic pathways |
|  | 4-Hydroxy-2-oxopentanoate | Organic acids | 6.395162 | Phenylalanine metabolism, Metabolic pathways |
|  | 2-Methylsuccinic acid | Organic acids | 6.395162 | - |
|  | Cucumegastigmane II | Sesquiterpenoids | 6.346252 | - |
|  | Ile-Gly-Val | Amino acids and derivatives | 6.244117 | - |
|  | 2-oxononadecanoic acid | Free fatty acids | 6.216185 | - |
|  | γ-Glu-Phe | Amino acids and derivatives | 6.198671 | - |
|  | LysoPC 15:0(2n isomer)* | Lipids | 6.162272 | - |
|  | 4,5,6-Trihydroxy-2-cyclohexen-1-ylideneacetonitrile | Alkaloids | 6.148006 | - |
|  | Geldanamycin | Alkaloids | 6.080232 | - |
|  | Ethylmalonic acid | Organic acids | 5.993742 | - |
|  | Jionoside D | Phenolic acids | 5.912053 | - |

| **TableS2. qRT-PCR primers used in this study** | |
| --- | --- |
| Name | Sequence (5' to 3') |
| HdαCA1-F | CAGCCGATGAAGAAGTTGAGG |
| HdαCA1-R | TGAGCGTGCCCAAATGAGAC |
| HdαCA2-F | GTCGCTTACCACGCCTCCTT |
| HdαCA2-R | CGTTTATTGCTTGAATGGGTC |
| HdαCA3-F | GGGAGTGGTGGAATGGCGATGT |
| HdαCA3-R | CCCTGGCTTGCCGTTGA |
| HdαCA4-F | TTCGGATGATGCCAAAGTTATT |
| HdαCA4-R | GGGAACACTGGAAGAGCCTAC |
| HdαCA5-F | CATTCCATCCCACAGAAGTCA |
| HdαCA5-R | CAAGGTCGGGCATTGTTCT |
| HdαCA6-F | GTCGTCATCCTGCCTACACTTGG |
| HdαCA6-R | CACCTCCTGCGTCTCCTTGC |
| HdαCA7-F | ACAGAGTCGTCGTCTTGCCTAA |
| HdαCA7-R | TCTGATGAACCAAGTGAACCTCC |
| HdαCA8-F | GAGTGGTATCACCCGCATTT |
| HdαCA8-R | TGGCTTCTTCCCGTCCC |
| HdαCA9-F | ACGGGAAGAAGCCAGTCG |
| HdαCA9-R | GCTCGTATTGCGTTTATTTGTTGT |
| HdαCA10-F | GACCATCAGACCCTTTCCTC |
| HdαCA10-R | ATCAACACTATCCCGCACCA |
| HdαCA11-F | TTCGCCAAAGCAAATAAAGG |
| HdαCA11-R | ATGATGAAACAAACCGTCCA |
| HdαCA12-F | GTATTACAGGTACATCGGTTCACTCA |
| HdαCA12-R | TTATTTGCTCCACGGACACG |
| HdαCA13-F | AAGTATTACAGGTACATCGGTTCG |
| HdαCA13-R | TTATTTGCTCCACGGACACG |
| HdαCA14-F | CACTGGCACTCGCCTTCTGA |
| HdαCA14-R | TCGCCAGGAGTTTGGACAGG |
| HdβCA1-F | GCTTCCTCACCTCACCCACTCT |
| HdβCA1-R | GCTTGGCAATGGATTCCTCGT |
| HdβCA2-F | TCCCGAGTATGCCCTTCCCACG |
| HdβCA2-R | GAAGTGGACCCATCATCAGGAA |
| HdβCA3-F | TCCTCCTTGTTTGCCTTCTTT |
| HdβCA3-R | TCCTCTTGTTATCCGTAATCTGC |
| HdβCA4-F | TCCTCCCTACGACAAGACCAAAT |
| HdβCA4-R | GCCTTACAAACGGATACGACAGAA |
| HdβCA5-F | TTCAGGAGTCGGTGCCGCTAT |
| HdβCA5-R | GCAGGACCATCATCGGGAGC |
| HdγCA1-F | CGAACGCAAGTGGGAAAGTG |
| HdγCA1-R | CGCAGATTGGGAGATGAAGG |
| HdγCA2-F | GGATGCGTTCTTAGAGGTGATG |
| HdγCA2-R | GGGCTCCAGCAGCAACCATT |

| **TableS3. Proteins used in this study** | | |
| --- | --- | --- |
| Name | GeneID | Species |
| AtαCA1 | AT3G52720.1 | *Arabidopsis thaliana* |
| AtαCA2 | AT2G28210.1 | *Arabidopsis thaliana* |
| AtαCA3 | AT5G04180.1 | *Arabidopsis thaliana* |
| AtαCA4 | AT4G20990.1 | *Arabidopsis thaliana* |
| AtαCA5 | AT1G08065.1 | *Arabidopsis thaliana* |
| AtαCA6 | AT4G21000.1 | *Arabidopsis thaliana* |
| AtαCA7 | AT1G08080.1 | *Arabidopsis thaliana* |
| AtαCA8 | AT5G56330.1 | *Arabidopsis thaliana* |
| AtβCA1 | AT3G01500.2 | *Arabidopsis thaliana* |
| AtβCA2 | AT5G14740.1 | *Arabidopsis thaliana* |
| AtβCA3 | AT1G23730.1 | *Arabidopsis thaliana* |
| AtβCA4 | AT1G70410.2 | *Arabidopsis thaliana* |
| AtβCA5 | AT4G33580.2 | *Arabidopsis thaliana* |
| AtβCA6 | AT1G58180.2 | *Arabidopsis thaliana* |
| AtγCA1 | AT1G19580.1 | *Arabidopsis thaliana* |
| AtγCA2 | AT1G47260.1 | *Arabidopsis thaliana* |
| AtγCA3 | AT5G66510.2 | *Arabidopsis thaliana* |
| AtγCAL1 | AT5G63510.2 | *Arabidopsis thaliana* |
| AtγCAL2 | AT3G48680.1 | *Arabidopsis thaliana* |
| HdαCA1 | Hdi011693 | *Hygrophila difformis* |
| HdαCA2 | Hdi011694 | *Hygrophila difformis* |
| HdαCA3 | Hdi012310 | *Hygrophila difformis* |
| HdαCA4 | Hdi012338 | *Hygrophila difformis* |
| HdαCA5 | Hdi023524 | *Hygrophila difformis* |
| HdαCA6 | Hdi024286 | *Hygrophila difformis* |
| HdαCA7 | Hdi024287 | *Hygrophila difformis* |
| HdαCA8 | Hdi028772 | *Hygrophila difformis* |
| HdαCA9 | Hdi028773 | *Hygrophila difformis* |
| HdαCA10 | Hdi028775 | *Hygrophila difformis* |
| HdαCA11 | Hdi028777 | *Hygrophila difformis* |
| HdαCA12 | Hdi028779 | *Hygrophila difformis* |
| HdαCA13 | Hdi028782 | *Hygrophila difformis* |
| HdαCA14 | Hdi032772 | *Hygrophila difformis* |
| HdβCA1 | Hdi000315 | *Hygrophila difformis* |
| HdβCA2 | Hdi004481 | *Hygrophila difformis* |
| HdβCA3 | Hdi010336 | *Hygrophila difformis* |
| HdβCA4 | Hdi019491 | *Hygrophila difformis* |
| HdβCA5 | Hdi028951 | *Hygrophila difformis* |
| HdγCA1 | Hdi005052 | *Hygrophila difformis* |
| HdγCA2 | Hdi026602 | *Hygrophila difformis* |

| **Table S4. Characteristics of CA genes in *H. difformis*** | | | | |  |  |
| --- | --- | --- | --- | --- | --- | --- |
| **Gene name** | **Gene ID** | **Length** | **Mw** | **pI** | **GRAVY** | **Predicted Subcellular localization** |
| HdαCA1 | Hdi011693 | 218 | 25404.58 | 6.45 | -0.607 | Cytoplasmic |
| HdαCA2 | Hdi011694 | 274 | 31457.87 | 6.95 | -0.483 | Cytoplasmic |
| HdαCA3 | Hdi012310 | 238 | 25313.76 | 8.76 | -0.206 | Extracellular; Periplasmic; Outer Membrane |
| HdαCA4 | Hdi012338 | 246 | 27341.42 | 9.25 | -0.376 | Periplasmic |
| HdαCA5 | Hdi023524 | 311 | 35369.39 | 9.36 | -0.456 | Periplasmic |
| HdαCA6 | Hdi024286 | 314 | 35056.4 | 9.74 | -0.278 | Periplasmic |
| HdαCA7 | Hdi024287 | 245 | 28034.73 | 5.75 | -0.658 | Cytoplasmic |
| HdαCA8 | Hdi028772 | 361 | 41025.72 | 8.6 | -0.552 | Periplasmic; Extracellular; Cytoplasmic |
| HdαCA9 | Hdi028773 | 169 | 18556.17 | 6.82 | -0.234 | Periplasmic |
| HdαCA10 | Hdi028775 | 184 | 20604.72 | 7.88 | 0.061 | Periplasmic; Outer Membrane; Cytoplasmic |
| HdαCA11 | Hdi028777 | 282 | 31668.09 | 6.65 | -0.234 | Outer Membrane; Cytoplasmic; Extracellular |
| HdαCA12 | Hdi028779 | 280 | 31766.3 | 9.12 | -0.466 | Cytoplasmic; Periplasmic |
| HdαCA13 | Hdi028782 | 280 | 31782.29 | 9.12 | -0.475 | Cytoplasmic; Periplasmic |
| HdαCA14 | Hdi032772 | 272 | 30995.39 | 8.66 | -0.54 | Cytoplasmic |
| HdβCA1 | Hdi000315 | 324 | 34798.9 | 7.56 | 0.01 | Periplasmic |
| HdβCA2 | Hdi004481 | 259 | 28549.95 | 5.95 | -0.177 | Cytoplasmic |
| HdβCA3 | Hdi010336 | 304 | 33832.44 | 7.1 | -0.295 | Cytoplasmic; Outer Membrane |
| HdβCA4 | Hdi019491 | 332 | 35426.69 | 6.66 | -0.027 | Periplasmic |
| HdβCA5 | Hdi028951 | 283 | 30688.75 | 8.38 | -0.119 | Periplasmic |
| HdγCA1 | Hdi005052 | 272 | 29620.62 | 5.99 | -0.143 | Cytoplasmic |
| HdγCA2 | Hdi026602 | 266 | 28438.32 | 6.08 | -0.037 | Cytoplasmic |
